# Optical and Visual Diet in Myopia

**DOI:** 10.1167/iovs.66.7.3

**Published:** 2025-06-05

**Authors:** Susana Marcos

**Affiliations:** 1 Center for Visual Science, Flaum Eye Institute, The Institute of Optics, University of Rochester, Rochester, New York, United States

**Keywords:** myopia, eye models, optical blur, environment

## Abstract

The alarming increase in myopia prevalence in modern times is attributed to environmental changes affecting young individuals. Visual scenes are projected onto the retina by the eye's optical components and sampled by retinal photoreceptors, shaping the spatial, temporal, and chromatic “visual diet” fed to the visual system. These inputs provide essential signaling for proper emmetropization and, in eyes that develop myopia, trigger the cascade of events leading to excessive axial elongation. This article offers foundational components for formulating computational models of myopic eyes and highlights available and needed optical and structural data to construct longitudinal three-dimensional optical eye models in emmetropes and developing myopes. These wide-angle eye models, in both relaxed and accommodated states, will enable understanding of the changes the eye undergoes at myopia onset and potentially allow exploration of cause–effect relationships in myopia development. Age- and refractive-dependent eye models also serve as platforms to test the coupling of novel optical treatments for myopia control with the resulting blur patterns across the retina. Chromatic, spatial, and temporal stimuli are explored as plausible cues for emmetropization. The article also reviews published theories on mechanisms for encoding the sign of defocus and triggering axial elongation. Fully quantitative technologies for ocular geometrical, biometric, and optical evaluation, as well as for monitoring physical features of the environment, are critical for collecting multidimensional data sets that enable predictive models. Given that time spent outdoors is a major factor associated with myopia development, exploring mechanisms that connect light exposure with myopia is paramount. The article also reviews current proposed mechanisms linked to retinal dopaminergic pathways, dysfunction of melanopsin signaling, and disruption of circadian rhythms—factors altered in modern lifestyles by artificial illumination and prolonged use of digital displays. Understanding the interplay between distinct environmental attributes (e.g., light intensity, spatial frequency distribution, blur, spectral characteristics) and the filtering effects of ocular optics will help elucidate pivotal myopiagenic signals and potentially unveil the complex, multifactorial mechanisms relevant for myopia management. Additionally, an annex appended to the article discusses unresolved inquiries and promising research directions in myopia research, drawing from expert insights.

The optics of the eye projects the image of the outside world onto the retina, ultimately determining the amount (and sign) of blur, distortion, and spectral content available for further stages in the visual system. Retinal image quality is determined by the morphology and structure of the cornea and crystalline lens, and multiple studies have explored (sometimes with conflicting results) differences in the optical aberrations and other geometrical attributes of the myopic eyes in comparison with emmetropic or hyperopic eyes.

Even when myopic and emmetropes and hyperopic eyes show differences, it is challenging to determine whether those differences are a cause or a consequence of axial elongation. Regardless of the case, optical changes in the myopic eye and the combination of the optics of the eye with that imposed by the correcting elements of myopia can be critical in determining the quality of the images (central and peripheral) that the eye is permanently exposed to. Furthermore, the optics of the eye is not static but dynamically changing with accommodation. Retinal image quality also varies with pupil diameter. Given the demonstrated connection of near work and/or light levels with myopia development, understanding those changes and potential differences between emmetropes and myopes in aberrations, accommodative response, and pupil is certainly important. Furthermore, with the peripheral retina identified as playing a role in eye growth control, wide-field eye models should be considered.

The advent of more accurate technologies to measure eye biometry, optical aberrations, cornea and lens geometry, accommodation, and peripheral optics is key to the development of eye models that relate optics and geometry in relation to refractive error and allow exploring the coupling of optics of the eye with myopia control techniques, as well as of potential biomarkers for myopia development and prospective success of myopia control alternatives.

## Geometry and Optics of the Myopic Human Eye

### Dimensions of the Myopic Eye

**Figure 1.**
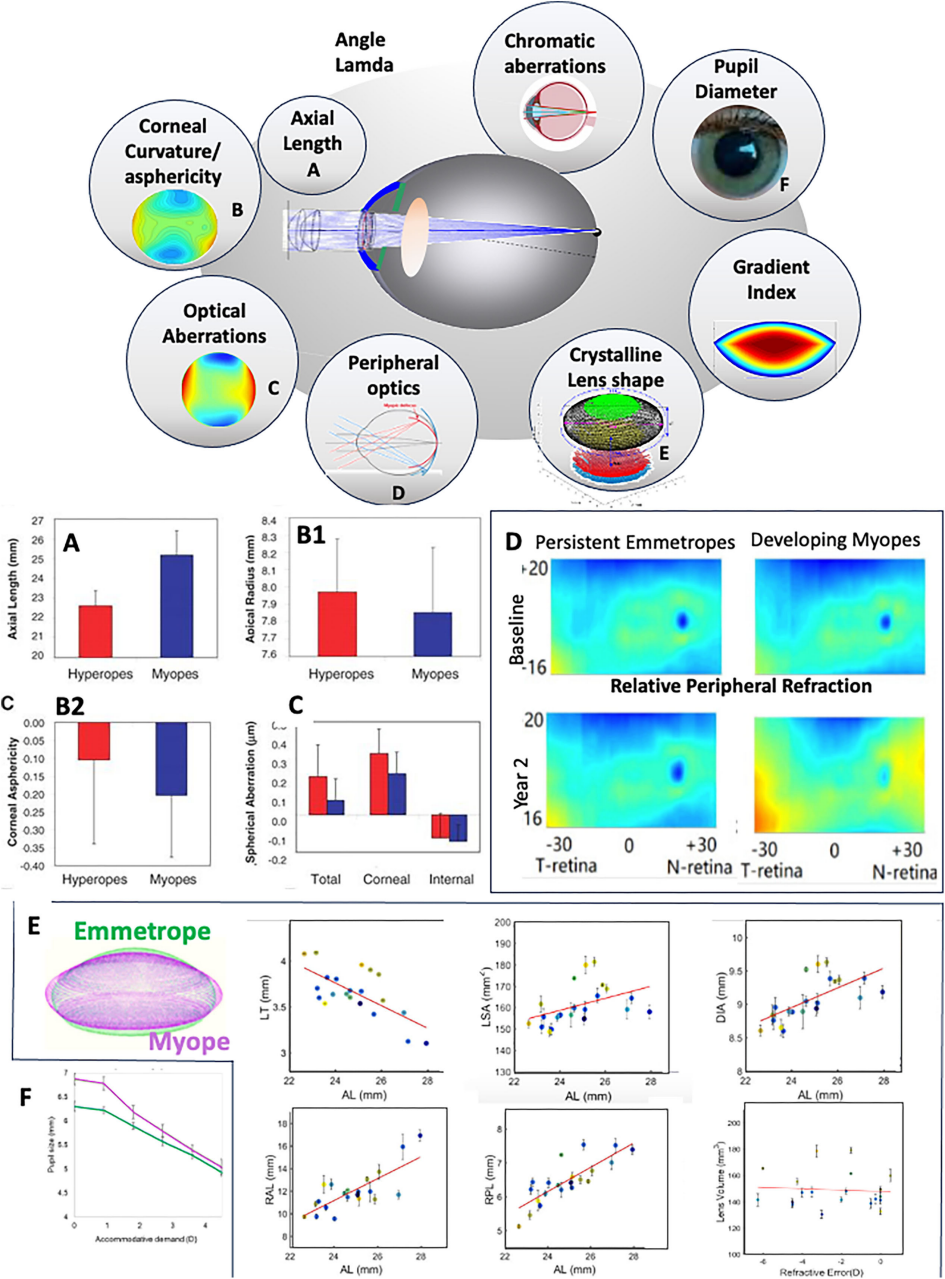
Optics and geometry of the myopic eye. *Upper*: Optical and geometrical properties that contribute to retinal image quality. Counterclockwise: Axial length; corneal radius of curvature and asphericity; optical aberrations; peripheral shape and optics; crystalline lens shape; Lens Gradient Index of refraction; chromatic aberrations; angle lambda (off-axis foveal position). Central image represents a computer eye model, which can be built for a specific patient using state-of-the-art technologies, most notably quantitative three-dimensional OCT. *Lower*: (**A**) Axial length (from low-coherence interferometry). (**B1**) Corneal radius of curvature. (**B2**) Corneal asphericity (calculated from corneal elevation maps). (**C**) Total eye, corneal and internal (by subtraction) spherical aberration (from corneal aberrometry and laser ray tracing aberrometry), in an age-matched and similar average magnitude of refractive error, in myopes (*blue*) and hyperopes (*red*), adapted from Llorente et al.^3^ (**D**) Two-dimensional maps of central and peripheral refractive errors at baseline (*upper*) and after 2 years (*bottom*), in persistent myopic children (*left*) and children who developed myopia, selected from Lin et al.^54^ (**E**) Cross-sectional crystalline lens parameters from three-dimensional quantitative anterior segment imaging and eigenlens representation, as a function of axial length (AL) or refractive error. *Upper panel left to right*: Lens thickness (LT), lens surface area (LSA), and equatorial diameter (DIA). *Lower panel left to right*: Anterior lens radius (RAL), posterior lens radius (RAL), and lens volume (VOL). *Left inset*: Superposition of reconstructed lenses in vivo from an emmetropic eye (*green*) and a myopic eye (*magenta*). The dependence of lens parameters on axial length/refractive error indicates lens stretching in the myopic eye. Data from Muralidharan et al.^18^ (**F**) Pupil diameter in myopic and emmetropic eyes while accommodating a high-contrast target (50 cd/m^2^) in an adaptive optics system. Adapted from Aissati et al.^27^

Retinal image blur is determined by the optical and geometrical properties of the ocular components that project the images of the outside world onto the retina. These parameters (summarized in Figure 1, upper panel) differ in myopes, in most cases as a result of myopia progression.

#### Axial Length

Without any doubt, the more salient difference in myopic eyes in comparison to emmetropes and hyperopes is their longer axial length^1^^–^^3^ (Fig. 1A). A recent meta-analysis revising data from >60,000 myopic eyes (49 studies, the majority in China) may be the most comprehensive report (in children and adolescents) of axial length and myopia, with a mean difference of 1.139 mm between myopes and emmetropes.^4^

#### Corneal Radius of Curvature

Several studies report steeper central corneas in myopes.^5^^,^^6^ Others do not report significant correlations between corneal curvature and refractive error.^7^ Conversely, some literature reports flatter corneas in longer eyes, with an increase in corneal radius (or decrease in corneal curvature) as axial length increases,^8^ which was hypothesized to result from the active emmetropization effect to compensate axial elongation. More recent results, using optical coherence tomography (OCT)-based corneal topography, also show flatter corneas, particularly in high myopia, but with a nonuniform correlation between corneal curvature and axial length, and uncorrelated in the longer eyes.^9^ The large meta-analysis by Zhang et al.^4^ reports corneal curvature from 9 studies and corneal radius of curvature from 12 studies (>5000 emmetropes and >10,000 myopes) and concludes that the myopic group has a larger average corneal curvature (0.253 D) or lower average corneal radius (−0.046 mm). Differences across studies about the association between corneal curvature and myopia/axial length may be related to differences in refractive error and axial length ranges in the studies, age range, differences in the way that corneal curvature is measured (technology and whether it is measured centrally or more peripherally), or ethnicity^10^ in the studies. It appears that neither steep nor flat corneas are strongly characteristic of myopes and that corneal shape could instead be associated with overall eye size in all dimensions.^11^ Figure 1B1 shows results from Llorente et al.^3^ Two take-home messages can be extracted from these findings: (1) when constructing a “myopic” eye model, corneal radius of curvature can be highly variable and probably related to overall eye size, not only axial length, and (2) clinically, it is critical to measure both axial length and corneal curvature, as flatter corneas in not very high myopes may hide long axial lengths. These differences may be also relevant in myopia correction/control techniques that rely on reshaping the corneal curvature (i.e., orthokeratology).

#### Corneal Asphericity

Cross-sectional^5^ and longitudinal^12^ studies report correlations between asphericity (Q) and myopia, with high myopes showing less negative or even positive asphericity, indicative of a decreased peripheral corneal flattening with increasing axial length. Conversely, Budak et al.^13^ reported more positive Q values in moderately myopic eyes and more negative Q values in high myopic eyes, with the highest association between myopic refraction and negative asphericity found in steeper corneas. It has been suggested that separate analysis of surface radius of curvature and asphericity, including correlations between both, can lead to overinterpretations of those features in relation to development, growth, or aging, while instead, those are a consequence of the fit.^14^ Alternative representations are likely more robust in an age-matched and absolute spherical error-matched comparison between myopes and hyperopes. Llorente et al.^3^ (Fig. 1B2) found a trend for myopic corneas to be steeper and to show more negative asphericities than hyperopic corneas (with high intersubject variability), but corneal spherical aberration (resulting from the combined effect of radius of curvature and asphericity) was statistically significantly lower in myopes than hyperopes. Despite differences across the studies, it may be safe to conclude that significant differences in shape/spherical aberrations across groups may be associated to ocular growth, with moderate hyperopic eyes being smaller^15^ and more spherical in overall shape, whereas moderate myopic eyes may flatten more in the periphery than in the central cornea as they elongate.

#### Crystalline Lens Shape

The crystalline lens continuously grows with age, and its age-related changes have been investigated thoroughly in vivo and ex vivo. However, full geometry of the crystalline lens has been little investigated in myopia. Garner et al.^16^ found lower power in the crystalline lens in myopes than emmetropes, and several reports indicate thinner lenses in myopes. Mutti et al.^17^ reported in a longitudinal study in children that myopic subjects exhibited thinner crystalline lenses (as well as lower lens power compared to emmetropic subjects). The recent meta-analysis by Zhang et al.^4^ in children and adolescents showed significantly thinner lenses in high myopes compared to emmetropes (by 0.046 mm, on average), based on results from nine studies (>1000 subjects in each group). Muralidharan et al.^18^ reported crystalline lens full shape parameters as a function of refractive error (and axial length) in myopic young adults, showing a statistically significant decrease in lens thickness (−0.12 mm/mm) and lens power (−1.2 D/mm) and an increase in lens equatorial diameter (0.15 mm/mm), posterior radius of curvature (0.36 mm/mm), and lens surface area (2.82 mm^2^/mm), while volume did not show a statistical with axial length or refractive error (Fig. 1D).

#### Crystalline Lens Gradient Index

The crystalline lens of many species, including human, varies from the nucleus to the cortex, conferring several advantages, including increased effective power, negative spherical aberration that helps to compensate for the spherical aberration of the cornea, and decreased back reflectance in the surfaces. In young lenses, the lens gradient index (GRIN) profile is parabolic, although with increasing age, the GRIN flattens, losing its compensatory role^19^^,^^20^ and playing a role in the change in lens power with age. While the lens GRIN has been thoroughly studied as a function of age and accommodation, to our knowledge, GRIN has not been investigated in relation to myopia in human eyes, except for a study in myopia animal models (guinea pig).^21^ Given the impact of GRIN in the aberrations of young eyes and its expected role in peripheral optics, modeling potential differences in the GRIN and GRIN contributions between myopes and emmetropes would help produce more accurate eye models.

#### Angle Lambda

The angle lambda is the angle between the pupillary axis and the line of sight, resulting from the off-axis position of the fovea. It is fairly well established that myopes exhibit a lower angle lambda than myopes and hyperopes.^22^ This is consistent with a constant physical displacement of the fovea from the “optical axis” of the ocular components in longer eyes.

#### Pupil Diameter

Pupil diameter responds to ambient light levels, changes with accommodation, and determines retinal image quality (through the relative contribution of diffraction and optical aberrations); therefore, it is an important factor to consider when studying both optical blur and retinal illuminance “diet” that the eye is subject to. Research on the relationship between refractive error and pupil diameter has yielded mixed results. Some studies have not found differences across refractive groups.^23^ A recent study in preschoolers found smaller and more variable pupils in myopes,^24^ while several studies have identified larger pupils in myopes.^25^^,^^26^ Figure 1F shows larger pupils in myopes than emmetropes at both relaxed and accommodated states.^27^

#### Optical Aberrations in Myopia

The role of axial aberrations in myopia development has been debated for almost two decades^28^^,^^29^ (see recent review by Gomes et al.^30^). On the one hand, aberrations alter retinal image quality and may modulate the accommodative lag and the signals for emmetropization. On the other hand, optical aberrations are a consequence of eye geometry, and morphologic changes in myopia may result in differences in the aberration patterns. Although some research has shown a correlation between myopia and increased optical aberrations,^31^^,^^32^ other work has found no correlation between refractive error and aberrations.^33^ A recurrent finding in many studies is the significantly lower amount of positive fourth-order spherical aberration in myopes.^3^^,^^29^^,^^34^^–^^36^ The lower corneal and total aberrations appear to be associated with a more prolate corneal shape^3^ (Fig. 1C) in myopes. Beyond spherical aberration and the actual shape of the cornea and crystalline lens, other factors may contribute to potential differences in other higher-order aberrations, including the relative tilt between lenses and the off-axis position of the fovea. For example, the reported balance between cornea and lens optics^37^ results in compensation of the lateral coma through a passive mechanism,^38^ potentially explaining the lack of differences in horizontal asymmetric aberrations between refractive groups. Some studies point out an increase in vertical coma in myopes,^32^ which could be related to the topographic changes induced by sustained reading potentially induced by the force of the eyelids.^39^ Studying the link between geometrical/structural features of the eye and the optical aberrations allows understanding the factors that contribute to optical degradation.

#### Chromatic Aberrations

Chromatic aberrations arise from the dispersion of the interocular media. Longitudinal chromatic aberration (LCA) is defined as the chromatic difference of focus between the two ends of the visible spectrum (around 2 D), with blue wavelengths focusing in front of the retina and red wavelengths behind the retina in an emmetropic eye (see Thibos et al.^40^ for a compilation of early research and Vinas et al.^41^ for objective and subjective measurements of LCA). Transverse chromatic aberration (TCA) arises from misalignment and optical imperfections and results in a lateral displacement of the image as a function of focus (see Rynders et al.^42^ for a TCA study on a large cohort). TCA appears to be much more variable than LCA across the population. Different studies have explored interactions between LCA, TCA, and monochromatic aberrations^43^^,^^44^ and the impact of chromatic and monochromatic aberrations (or their correction) on visual quality.^45^^,^^46^ One study measured LCA in infants,^47^ and expectedly, due to its higher optical power, found it higher than in adults. Another study measured LCA in emmetropic and myopic adults^48^ and found that despite some variability in LCA in the population, this was unrelated to refractive error.

#### Peripheral Retinal Shape, Refraction, and Aberrations

A role of the peripheral retina is often invoked in the development of myopia, with peripheral (rather than central) hyperopic defocus identified as a trigger for myopia. Of course, differences in retinal shape (leading to differences in peripheral refractive states) between emmetropes and myopes could also be a consequence rather than a cause for axial elongation. Verkicharla et al.^49^ and, more recently, Matsumura et al.^50^ have published comprehensive reviews of studies measuring total eye shape and retinal eye shapes, often described by conics, or alternatively ratios between axial length (AL), height (H), and width (W), as well as relations between retinal shape and peripheral refraction and aberrations. Except for the very marked higher AL than W and H in myopes than emmetropes, differences in eye shape across refractive groups do not appear drastically different, with a large variability across individuals within similar refractive groups.^51^ Around 25% of the examined eyes by Atchison et al.^51^ fitted global expansion models (myopic eyes larger in all dimensions) and axial expansion models. This study^51^ (and another by Gilmartin et al.^52^) found oblate shape retinas in most eyes, but less so in myopic eyes, as well as higher asymmetries of the posterior retinal shape (more steepening in the transverse axial direction than in the sagittal direction). Tabernero and Schaeffel^53^ also found more irregularities in retinal shape in myopic eyes. A longitudinal study in a cohort of 214 children in Hunan Province in China shows little difference between two-dimensional peripheral refraction maps at baseline between emmetropic children who remained emmetropic and those who developed myopia during the study period (1–2 years).^54^ However, myopic children showed relative hyperopia, particularly in the horizontal meridian^54^ (Fig. 1D).

Because peripheral optics depends also on the contributions of the cornea and crystalline lens, and these vary across refractive groups (see above sections), inferring peripheral eye length from refraction may lead to artifactual conclusions. Again, the literature is extensive on peripheral refraction. In general, it appears that myopic patients have less myopia peripherally than centrally,^55^ although the differences in peripheral refraction between myopes and emmetropes could be restricted to some specific areas, in particular the temporal retina.^56^ Kang et al.^57^ observed differences in peripheral refraction across ethnicities, with East Asians exhibiting a greater degree of relative peripheral hyperopia than white subjects. Differences in retinal shape may also have an impact on peripheral astigmatism. For example, Queirós et al.^56^ reported nasal-temporal asymmetries in the astigmatism J180 component in myopes, along the horizontal visual field. Also, in the vertical visual field, J45 changed at three times the rate of change in the horizontal visual field with increasing myopia. Osuagwu et al.^58^ also found considerable nasal-temporal asymmetry in peripheral refraction in both groups and superior-inferior asymmetry for hyperopes but little difference between groups in astigmatic components or asymmetric higher-order Zernike coefficients.

#### Cone Spacing, Ratios, and Photopigment

Although not technically part of a computer eye model, the sampling of the retinal image by the retinal mosaic is the immediate step following optical imaging. Several studies have measured cone spacing in human fovea and/or parafovea from excised retinas and in vivo, most recently using Adaptive Optics (see Wang et al.^59^ for a recent literature review and a detailed analysis on data on cone spacing as a function of axial length). With increasing axial length, foveal cone interspacing expands, but at a lower rate than eye length. As a result, although retinal stretching occurs in myopes (decrease in cones/mm^2^), they tend to show a higher angular sampling density (in cones/deg^2^). These results favor models predicting global expansion but also foveal overdevelopment (cones more spread out in the fovea of myopes). Literature using ERG-flicker photometry methods reports associations between long/middle wavelength (L/M) ratio and ethnicity and refractive error, with higher L/M ratios in Caucasian emmetropic populations,^60^ suggesting a possible protective role of a higher percentage of L cones against myopia.^61^ As one would expect L/M cone ratios in humans to be correlated with L/M cone sensitivity,^62^ this finding is in contradiction with reports of a higher L cone sensitivity correlated with myopia.^63^ The latter was attributed to a higher luminance sensitivity in the L cones, possibly as a result of those being more strongly stimulated by images behind the retina and triggering axial elongation to maximize L cone contrast. On the other hand, the potential association of a higher density or sensitivity for a given cone class with myopia does not appear supported by the reported shorter eyes found both in protanopia and deuteranopia.^64^ Remarkably, blue cone monochromacy is generally associated with high myopia, although the variability is large (including hyperopia), and the association is most likely the result of the specific genetic mutations, which may affect both S-opsin expression and myopia.^65^ Recent work by Neitz et al.^66^ found a subset of haplotypes of the genes encoding L and M cone opsins that caused exon skipping (therefore reducing photopigment in subsets of cones) to be associated with myopia.

In conclusion, although there is large intersubject variability in the ocular dimensions and some contradictions in the literature, myopic eyes appear to have some distinct features (higher axial lengths, slightly steeper corneas, thinner, flatter and equatorially more stretched lenses, relatively more hyperopic peripheral retinas, larger pupils, lower angle lambda), indicating the need to build specific eye models for myopes.^33^ However, these computer eye models need to be age- and refraction-specific, and they require a large degree of coordination between ocular components to be realistic. Custom-built eye models^67^ based on accurate three-dimensional biometry will be able to simulate the images falling on the retina (on- and off-axis) to computationally evaluate the optical and visual diet available to the patient and to study the coupling of corrective devices (i.e., multifocal contact lenses, spectacles) with the eye's optical system via ray tracing.

### The Eye at Myopia Onset and During Myopia Progression

The eye component dimensions discussed in section 1.1 are generally obtained from cross-sectional studies and give a “fixed” picture of the eye model, most often in myopic young or older adults. While this information is important to better describe a myopic eye, “virtually” test the optical effect of different corrections (spectacles, contact lenses, intraocular lenses) in realistic myopia eye models, or even customize the corrections to the anatomy of the myopic eye, only longitudinal measurements will be able to inform the optical (and therefore retinal image quality) changes undergone by the eye during the development of myopia. Furthermore, longitudinal studies that include patients before they become myopic will give insights into early biomarkers for myopia development, retinal image quality exposure before the onset of myopia, and the potential for exploring the active or passive role of the crystalline lens in emmetropization and in the development of refractive errors. Ultimately, longitudinal computer eye models could re-create the anatomic and optical changes during eye growth and myopia development.

#### Longitudinal Changes in the Ocular Components

A small number of longitudinal studies have investigated ocular component growth curves in large cohorts of infants and school children, including the Orinda Longitudinal Study of Myopia^68^ and the Collaborative Longitudinal Evaluation of Ethnicity and Refractive Error (CLEERE)^69^ studies in the United States, the Singapore Cohort study Of the Risk factors for Myopia (SCORM),^70^^,^^71^ and several studies in China (most notably the Guangzhou Twin Study^72^) reporting the changes in spherical error, axial length, corneal and lens power, and, in some cases, lens thickness and other one-dimensional biometry data. It is well established that the cornea does not contribute actively to emmetropization or myopia development, stabilizing its curvature at 2 to 3 years of age.^73^^,^^74^ A longitudinal study in a Chinese population (7 to 12 years of age) showed steeper corneas in persistent myopes and flatter corneas in persistent hyperopes and emmetropes but no change with age,^75^ in good agreement with earlier work in a more ethnically mixed population.^76^ In contrast, axial length and the crystalline lens undergo drastic longitudinal changes during the critical period of myopia development, as confirmed (with subtle differences) by the abovementioned longitudinal studies. In particular, eyes that eventually become myopic experience a rapid decrease of crystalline lens power and an increase in axial length, with those changes occurring before they officially become myopic,^70^ as well as in children who were already myopic. A recent longitudinal study^71^ that sorts out subjects by age of myopia onset reveals that the fastest rates of a decrease in lens power occur in developing myopes (around 8 to 10 years of age), particularly around a year before myopia onset, concurrent with faster axial growth and myopic shifts, compared to emmetropes. Interestingly, the significant acceleration in lens power loss appears to compensate for the higher axial growth, postponing the onset of myopia. The mechanisms for lens thinning and power loss and whether those result from an active or passive compensation are not well understood. While it is unlikely that changes in lens power drive continuous axial elongation, as the eye continues to grow, the mechanisms by which the lens presumably stretches to decrease power (more rapidly in developing myopes), ceasing at around age 10, are intriguing. Different hypotheses have been postulated, including equatorial lens stretching until physically possible^77^^,^^78^ or remodeling and compactification of the crystalline lens fibers leading to steepening of the gradient index.^76^

#### Longitudinal Changes in Peripheral Optics

It is fairly well established that as myopia progresses, the eyes become larger in all dimensions, but predominantly axially, followed by height and width.^51^ Mutti et al.^79^ followed a cohort of children for over a decade (5 to 15 years), and their measurements included peripheral refraction at 30° nasal. Myopia progression involved changes in relative peripheral refraction toward hyperopia, but at baseline, their peripheral refraction was unrelated to the risk of developing myopia.^80^ Similary, Sng et al.^81^ did not find differences in the peripheral refraction between children who became myopic and those who did not, as has been corroborated recently by Lin et al.^54^ using two-dimensional peripheral refraction maps, suggesting that the differences in peripheral refraction between myopes and hyperopes are a consequence rather than preceding myopia.

In conclusion, a number of changes occur during critical periods of myopia development, mostly involving the crystalline lens and eye shape. While many of those changes appear to be a consequence of axial elongation, the active or passive role of the crystalline lens is yet not known. Whether some particular geometrical features precede myopia onset or could be identified as a risk factor for myopia remains to be identified. Longitudinal model eyes incorporating information of the coordinated morphologic changes occurring during normal and myopic eye growth will shed light on the retinal image quality that eyes are exposed to before and during myopia development and can be ideally combined with prospective myopia control optical strategies to model the imposed changes in retinal image, both central and peripherally.

### Accommodation and Myopia

Near work has often been associated with a higher prevalence of myopia (see section 3 and Dutheil et al.^82^ for a recent review and meta-analysis). As with other components, there has been debate whether the potentially reduced accommodative response is a cause or a consequence of myopia.^83^ Understanding potential differences in the crystalline lens accommodative response between myopes and emmetropes is important, among other reasons, for the purposes of constructing accommodating eye models. Section 1.1 described associations between crystalline lens shape and refractive errors. Given that accommodation is accomplished by the crystalline lens, it would not be unlikely that those differences extend also to the accommodating lens. On the other hand, if myopes are poorer accommodators and expend more time performing near work, they would be exposed more permanently to hyperopic defocus, a trigger for axial elongation.

#### Accommodative Lag and Myopia

While there is no consensus whether accommodative lag is elevated or not before myopia onset, there are numerous reports indicating that myopes (both adults and children) have larger accommodative lags,^84^^–^^86^ with the accommodative response function decreasing as myopia progresses.^87^ Labhishetty et al.^88^ have suggested that this lag may be due to reduced accommodative sensory gain. On the other hand, recent work using binocular wavefront sensing technology to further explore accommodation dynamics did not find associations between refractive errors and dynamic accommodation parameters, such as accommodative speed and duration, but match the bulk of literature showing increased accommodative lag in myopes.^89^

#### Morphological Changes and Accommodation

Recent OCT-based quantitative tools have been applied to assess the full shape of the crystalline lens shape (anterior and posterior radii of curvature, equatorial diameter, thickness, surface area, volume, and eigenlens parameters) in myopia,^18^ as well as their change with accommodation.^90^^–^^92^ Studies could be expanded to compare the accommodating lens shape across refractive errors. A study by Bolz et al.^93^ looking at axial biometry during accommodation found larger increases in lens thickness and posterior pole position and a decrease in anterior chamber depth per diopter of accommodation in myopes compared to emmetropes, suggesting that larger lens reshaping is needed in myopes to achieve the same focus change. A recent study by Wang et al.^94^ also corroborated larger changes in the posterior lens in higher myopes. Mechanical models of the lens in young myopes based on realistic crystalline lens morphology as a function of accommodation could shed light on the relations between accommodative forces, lens reshaping, and power change.^95^ The morphology of another element of the accommodative plant, the ciliary muscle, has also been investigated in relation to refractive error during regular and sustained accommodation, using OCT.^96^^,^^97^ Myopes were found to exhibit smaller reductions in ciliary muscle thickness but larger muscle movements than emmetropes. Sustained accommodation (>30 minutes) reduced ciliary muscle thickness generally in all refractive groups, but differentially in amounts and regions, and only myopes developed a sustained myopic shift. Those differences in ciliary muscle morphology and response to sustained accommodation with refractive error raise the question of its potential involvement in the development of myopia and of the origin of those differences, which the authors point to as elevated activity of the sympathetic nervous system under sustained stress interstitial fluid changes.^96^ Again, longitudinal measurements could reveal preexisting conditions in myopes and/or a remodeling exacerbated by elevated near work activity.

Given that myopia arises from scleral remodeling that manifests anatomically by an increased scleral creep rate (i.e., scleral tissue elongation under a constant load, for example, IOP), some studies have pointed to potential correlations between IOP and axial growth (see Zhang et al.^98^ and Yii et al.^99^ for recent reviews on the matter). Even if there is still controversy on the association of IOP and myopia progression, and the underlying mechanisms are likely complex, it appears that accommodation could induce variations in IOP. Some studies specifically point to an increase in IOP with accommodation in progressing myopes and not in emmetropes.^100^

#### The Effects of Accommodation on Peripheral Optics

Given the potential role of both accommodation and peripheral optics in myopia, investigating peripheral refraction upon accommodation may be informative. However, the studies are inconclusive. Walker and Mutti^101^ and Whatham et al.^102^ found a hyperopic shift in relative peripheral hyperopia in accommodating eyes, while other studies^103^^,^^104^ did not find accommodation-related differences in relative peripheral refraction for either emmetropic or myopic groups. More recently, Queirós et al.^105^ reported that peripheral refraction in accommodating eyes (4 D) is not significantly different between emmetropes and myopes (with their spectacle corrections), and if anything, differences can only be accounted by peripheral astigmatism.

### Technologies to Assess Biometry and Optics

The assessment of optical and geometrical properties in relation to refractive error and the reliability of models based upon this information strongly rely on the accuracy of the instrumentation. The increased resolution of the imaging techniques and the advent of new modeling approaches have opened new opportunities for analysis, hypothesis testing, and the potential for evaluating new therapeutic interventions. Still, knowledge of the limitations, potential artifacts, and improvement strategies is needed for proper interpretation and model feeding.

#### Refraction and Aberrometry

Refractive error is used to categorize myopia and progression, but variations exist across techniques (objective and subjective) and whether measurements are collected with or without cyclopegia. Compared to cyclopegia, noncyclopegic measurements overestimate myopia.^106^ Also, under noncyclopegia, objective instruments tend to overestimate myopia compared to subjective refraction, although open-field autorefraction does so to a lower extent than photoretinoscopy and conventional autorefraction.^107^ Aberrometry has been recognized as the most suitable method to measure refraction, providing the closest values to subjective refraction.^108^ In particular, retinal image quality metrics consider interactions between low- and high-order aberrations, and refractive error can be obtained from the defocus (and astigmatism) that optimizes a given optical quality metric, for example, Strehl ratio^109^^–^^110^ or visual Strehl.^111^ As aberrometers generally work with infrared light, results should be corrected for LCA and the wavelength dependency of retinal reflectivity.^112^ Wavefront sensor–based see-through, binocular autorefractors have been shown to provide nonstatistically significantly different refractions from those obtained by subjective refraction, and they combine the benefits of aberrometry and open-field with a rapid acquisition and operator independency.^113^

#### Accommodation

Measurement of the accommodative response with increasing accommodative demand is particularly important when studying myopia, as a way to assess the blur to which subjects are exposed when performing near tasks and whether larger accommodative lags precede myopia or at least are larger in myopes. Kaphle et al.^114^ acknowledge differences between autorefractometry and aberrometry-based objective measurements of accommodative lags and attributed bias to autorefractor calibrations based on relaxed accommodation (not subject to negative spherical aberration in increased accommodative responses) and pupil mismatch between a natural pupil and that used by the instruments. Plainis et al.^115^ and Gambra et al.^90^ demonstrated significant shifts in the estimates of accommodative lags when high-order aberrations are incorporated. Labhishetty et al.^116^ compared the accommodative lag obtained from autorefractometry, aberrometry (residual defocus that maximized wavefront root mean square (RMS) or retinal image quality metrics), and subjective measurements based on maximization of acuity. Autorefractometry reported the largest lag, whereas visual performance measurements were maximized very close to the accommodative demand, therefore predicting low accommodative lag. Other factors that affect accommodation measurements are the presence or absence of convergence and proximity cues, luminance, spatial frequency, and spectral content of the stimulus and the level of cognitive demand for the task.

#### Axial Length

It is generally acknowledged in the clinical practice that the best method to diagnose myopia is measurement of refractive error, while myopia progression is best monitored by axial length measurements. Most techniques to measure axial length have been driven by the need of accurate biometry in the calculation of the optimal intraocular lens power in cataract surgery, and in state-of-the-art practice, ultrasound biometry has been almost entirely replaced by optical techniques, low- or partial-coherence interferometry (one-dimensional biometry), and. more recently, swept-source optical coherence tomography.^117^ The axial resolution of OCT is proportional to the square of the central wavelength of the illuminating source and inversely proportional to the spectral bandwidth of the source. For typical low/partial-coherence interferometers used in myopia research or practice, they range between 5 and 20 µm. For example, Lenstar 900 (Haag-Streit, Koritz, Switzerland), IOLMaster 500/700 (Zeiss, Jena, Germany), and Myopia Master (Oculus, Wetzlar, Germany) have peak wavelengths of 820, 780, and 880 nm, and bandwidths of 25 nm, 3 nm bandwidth (Zeiss), and not reported, respectively. Typical axial length repeatability of these devices is 0.01 mm, and differences between systems are 0.02 mm.^118^ OCT-based devices measure optical path lengths, which need to be divided by the mean group refractive index at the peak wavelength. Apart from calibration factors (in some cases against an ultrasound-based benchmark), variations in the refractive index across the different ocular media have been identified as a potential source of error.^119^

Although choroidal thickness has not been discussed in this article, as it is not expected to be included in optical modeling, it is nevertheless interesting to be mentioned in the context of biometry, as it has been recently used as a biomarker for defocus and to assess short-term responses to myopia control treatments. The choroidal physiology is complex, with numerous functions and responses to external stimuli. Variations of choroidal thickness in response to myopia induction or inhibitory signals could be mediated by its role in the production of all-*trans*-retinoic acid or growth factors involved in scleral remodeling.^120^ Choroidal thickness has been measured with low-coherence interferometry or OCT. However, the reported short-term variations in choroidal thickness (5 to 30 µm) are within the order of magnitude of the axial resolution of the technique, sometimes calculated indirectly (from axial length measurements) and challenged by superposition of other factors (light exposure, time of the day, etc.), eye movements, and difficulties in image segmentation (for a comprehensive review, see Ostrin et al.^121^). Technological improvements leading to an increase in the accuracy of choroidal thickness measurements (i.e., larger spectral bandwidth infrared (IR) sources, with sufficient penetration) are key to consolidating them as a biomarker for both understanding retinal signaling pathways in myopia and a clinical predictor for progression.

#### Retinal Shape and Peripheral Optics

Eye shape has been measured by magnetic resonance imaging and also partial-coherence interferometry (axial lengths determined at different eccentricities) and indirectly from peripheral refractions (assuming an eye model and changing the retinal curvature to match the measured refraction). In a technical note, Verkicharla et al.^122^ provide a comprehensive review of the assumptions and limitations of each technique.

Peripheral refraction and aberrations have also been measured through various techniques, including retinoscopy, photorefraction, autorefraction, laboratory-based or commercial Hartmann–Shack aberrometry, double-pass retinal imaging technique, and dedicated scanning aberrometry systems. Romashchenko et al.^123^ have presented a review and analysis of 16 studies reporting peripheral optics data with those different instruments. As pointed out by the authors, factors such as the effect of pupil shape and size, as well as the different conventions in reporting refractive errors, such as astigmatism, pose challenges in comparing the results and interpreting the data. As discussed in other sections, estimations of retinal image quality metrics appear to be the most suited to estimate the peripheral image quality across the visual field.

#### Crystalline Lens Shape, GRIN, and Ciliary Muscle

The crystalline lens power has been traditionally estimated from the difference between total refraction and corneal power. All optical techniques measuring directly crystalline lens shape (i.e., Purkinje-based phakometry, Scheimpflug imaging, or OCT) are subject to optical refraction from preceding surfaces, which needs to be counteracted to estimate accurate data. When those corrections are applied, a reasonable correspondence between lens radii of curvature is found.^124^ Magnetic resonance imaging has been applied to image cross sections of the crystalline lens,^125^ with the advantage of nonoptical distortions and complete lens visibility (not partially obstructed by the iris), although with the disadvantage of lower resolution compared to techniques such as OCT, slow acquisition, and high cost. Ortiz et al.^126^ applied fan (arising from the scanning architecture) and optical (arising from refraction) distortion corrections in OCT to provide fully quantitative three-dimensional images of the crystalline lens in vivo. An extrapolation method, trained on a database of ex vivo crystalline lenses,^127^ and a compact data basis for representation (eigenlens)^128^ allows estimations of the full lens shape (beyond the iris margins) with a high degree of accuracy from which parameters such as lens volume, surface area, or equatorial diameter can be obtained, as well as eigenlens coefficients, of which only five to six are needed to describe the full lens shape.

Direct estimates of lens GRIN in intact eyes have been obtained from magnetic resonance imaging measurements (ex vivo and in vivo),^129^^,^^130^ with the assumption that the variations in GRIN are associated with the concentration of crystalline proteins. Alternatively, OCT has been to estimate GRIN in intact crystalline lenses in vivo, assuming a certain parametric model of the crystalline lens profile and using measured lens surface shapes in two orientations.^131^ Comparisons of predicted power and spherical aberrations from ray-tracing simulations using measured lens surface and reconstructed GRIN and measured values of laser ray tracing reveal a good match between measurements and predictions,^132^ indicating that computer eye models can make use of OCT-based information to construct accurate eye models with realistic crystalline lens geometry and structure.

Ciliary muscle has been traditionally been visualized in vivo through ultrasound biomicroscopy, although the relatively low resolution, contact nature of the technique, and variations associated with the probe alignment make quantification challenging in patients. Ruggeri et al.^133^ presented a transscleral OCT operating at 1325 nm in synchronization with anterior segment imaging, allowing quantification of ciliary muscle volume and movement during accommodation.

#### Cone Spacing

Adaptive optics (AO), which corrects the aberrations of the eye, allowing imaging of the retina at a high resolution, is generally the technique of choice to visualize the cone photoreceptors (as well as other structures and retinal mosaics). Both scanning laser ophthalmoscopy and, more recently, OCT, combined with AO, have been used to measure cone spacing in human subjects in vivo (see Marcos et al.^134^ and Williams et al.^135^ for reviews in the field). Roorda and Williams^136^ used differential bleaching to identify the S, M, and L cone classes.

## Optical Blur: Trigger for Myopia and Sign Detection

**Figure 2.**
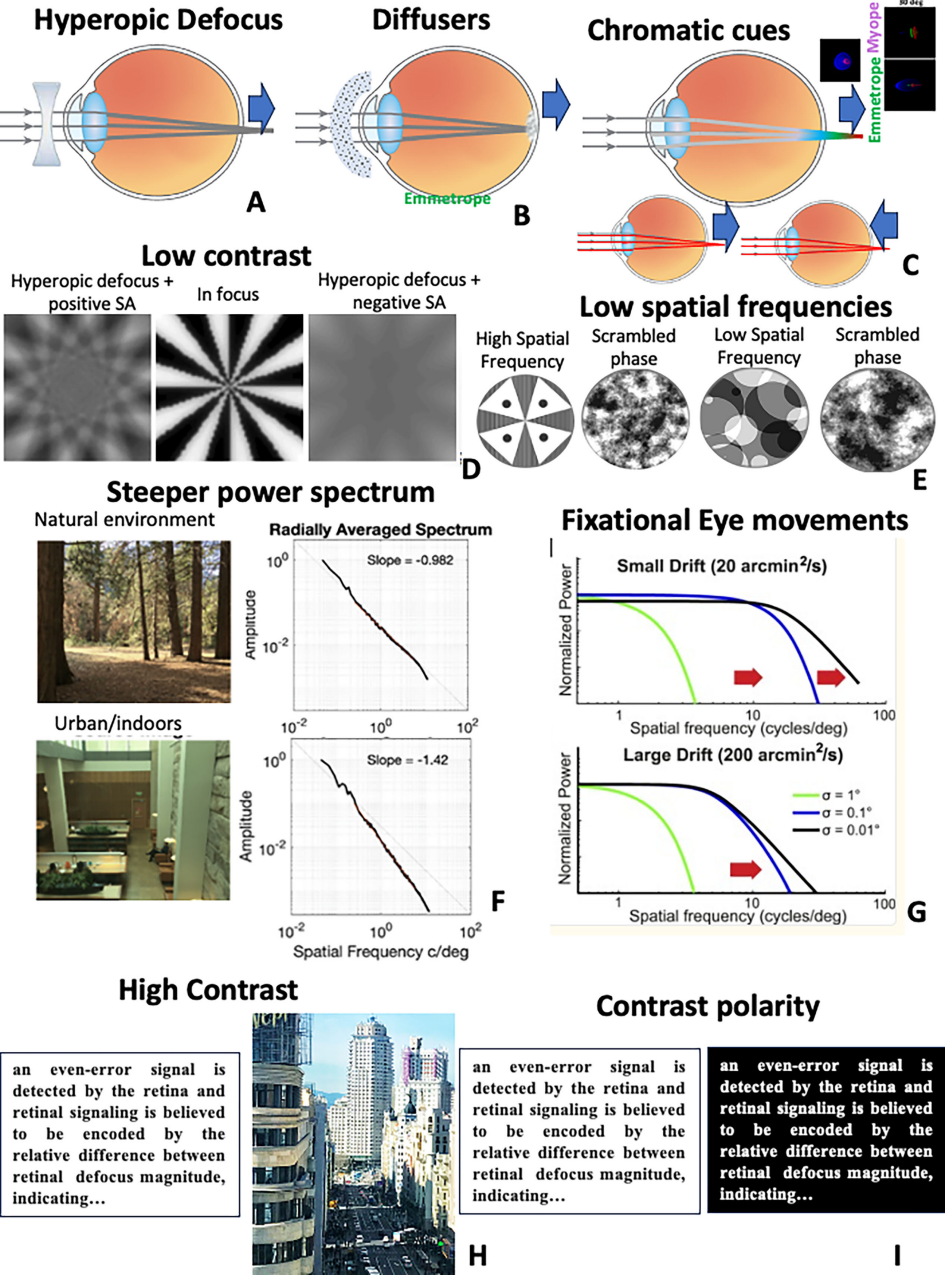
Myopia-triggering signals. (**A**) Retinal hyperopic defocus. An image formed behind the retina triggers axial elongation. (**B**) Form deprivation myopia. Diffuser googles placed in front of the eye reduce retinal image contrast (open loop), triggering axial elongation and myopia. A and B have been demonstrated in chickens, guinea pigs, tree shrews, and Rhesus monkeys, with some evidence in humans, as discussed in the text. (**C**) Chromatic cues. According to this theory, if the image is sharper in blue than in green and red, this sends the eye a signal to elongate; therefore, the sign of defocus may be encoded through the presence of LCA (Gawne et al.^158^) in the eye. In another theory, the blur anisotropy produced by a combination of defocus, astigmatism, and LCA off-axis differs between refractive groups (adapted from Zheleznyak et al.^164^). Narrow spectral illumination may cause myopia in red light (as found in chicken and guinea pigs) as the eye would elongate to meet the best focus in red, indicating proper emmetropization without needing LCA (*bottom left inset*). In other species (tree shrew, rhesus monkey), the opposite happens, with red light inhibiting axial elongation, suggesting the need of LCA for emmetropization (*bottom right inset*). (**D**) Combinations of defocus and spherical aberration can disambiguate if blur is positive or negative. This scenario occurs in accommodation, where hyperopic defocus arising from accommodative lag typically combines with negative spherical aberration (*right*), resulting in low contrast and a signal for axial elongation. Adding positive spherical aberration (*left*) increases contrast, reducing the myopigenic signal. Simulated images following the procedures described in Thibos et al.^156^ (**E**) The presence of high spatial frequencies (*left*) inhibits myopia, whereas lower spatial frequencies (*right*) trigger axial elongation. The effect remains even when presenting the same images with a scrambled phase (second and fourth images), indicating that the effect is driven by the absolute energy. Simulated images following the procedures described in Hess et al.^165^ for experiments in chickens. (**F**) Natural images exhibit 1/f frequency spectral slope. The spectral slope is steeper (reduced high spatial frequency content) in images of artificial objects or outdoor environments, which could be a trigger for myopia. Adapted from Flitcroft et al.^187^ (**G**) Changes in the temporal power spectra for three different levels of blur (indicated by σ) for small (*upper*) and large (*lower*) drifts. For sharper images, the range of frequency equalization expands, particularly for larger drifts. The sign of defocus could be estimated by comparing the amplitudes of temporal modulations, although this could vary in efficiency for different patterns of fixational eye movements. Data from Rucci and Victor.^169^ (**H**) In the high-contrast theory, high contrast such as that found in text at near or the nonnatural world would be triggers for axial elongation (see text for details). Photography by Susana Marcos. (**I**) In the contrast polarity theory, black text on white background would overstimulate the OFF retinal pathway, leading to myopia (see text for details).

A large body of literature, mostly from studies using animal models of myopia, indicates that retinal blur is a potent signal for modulating axial elongation. Two forms of blur consistently trigger myopia: negative lenses (this is a closed-loop signal, where the eye would grow axially to deliver a focused image when the ocular optics is combined with the negative lens, in other words, the eye would emmetropize to the new optics; Fig. 2A) and form deprivation (this is an open-loop signal, generally produced by diffusers placed in front of the eye; Fig. 2B). Authors have debated on the potential differences and similarities of the underlying mechanisms of myopia development from these two blur forms,^137^ as well as how applicable the findings in animal models, where myopia is induced with diffusers or lenses, are to human myopia.^138^^,^^139^ A fair argument is that the magnitudes of blur imposed to induce myopia in animal models are larger than those humans are possibly exposed to in a natural environment. Although animals are normally not spontaneously myopic, reports of marmosets (a nonhuman primate) developing modest or even severe myopia when raised in a laboratory environment^140^ point to possible effects of hyperopic blur imposed by the presence of only near targets or of lower lighting levels in an indoor environment. Interestingly, elevating lighting conditions appears to have a protective effect against myopia induction in form deprivation but not lens-induced myopia in primates (see She et al.^141^ for a relatively recent review).

Given the consistent effects of form deprivation and negative-lens defocus in inducing myopia in animals, the quest for analogous signals for myopia induction in humans is a natural step. The following section will focus on the effects of natural form deprivation conditions in humans (by, for example, congenital cataracts), as well as the effects of hyperopic defocus (either by spectacle corrections or accommodative lags) in the fovea or periphery. If those signals, as in the animal model literature, are triggers for myopia, then preventing or reverting them could be a strategy for myopia control.

Also inspired by the animal model literature, which shows differential modulation of eye axial length with negative and positive lenses (promoting or inhibiting axial elongation, respectively), efforts have been made to understand how the eye detects the sign of defocus to determine in which direction to grow. The following sections will revise the optical signals (many of them putatively arising from the optical features described in section 1) that could stimulate axial growth. As in the animal models fitted with negative lenses, myopia could result from a proper emmetropization to a closer environment. In alternative theories, myopic eyes would instead show an impairment in the sign of defocus detection mechanism.

### Form Deprivation and Hyperopic Defocus as Triggers for Myopia

#### Form Deprivation Myopia in Humans

Studies of form deprivation in infants and children are obviously restricted to deprivation occurring from natural causes, such as corneal opacification and congenital or traumatic cataract. Reports in pediatric patients with corneal opacification consistently show increased axial lengths and myopia, both when comparing a clear cornea group with a group with bilateral corneal scars (age- and sex-matched controls), as well as between the affected eye and the contralateral normal eye in patients with unilateral corneal scars.^142^ Reports on axial length in children with cataracts are more spread. While several studies point to longer eyes in the presence of the cataract,^143^ others show that the eye with cataracts was shorter than the normal fellow eye in children with unilateral congenital cataract.^144^ The findings may be complicated by the frequent comorbidity of microphthalmia and cataract, the potentially critical role of crystalline lens in emmetropization, and different age ranges across studies.

#### Effect of Negative Lenses (Overcorrection) in Humans

While there are several studies in the literature investigating the effect of undercorrection (less negative lenses) on myopia progression, offering equivocal results (see Logan and Wolffsohn^145^ for a systematic review of eight cohort studies), direct evaluations of overcorrection are expectedly lacking. Indirectly, overcorrection can be found in aphakic eyes (or pseudophakic eyes overcorrected as a result of challenging intraocular lens power selection) in children operated on for congenital or traumatic cataract. Although interpretation may be challenged by the abnormal nature of these eyes and the additional effects of lensectomy on eye growth,^146^ the literature shows increased axial length in aphakic eyes (presumably overcorrected, i.e., under-plus) and pseudophakic eyes compared to the contralateral normal eyes.^147^^,^^148^ In a clinical trial for treatment of exotropia, Chen et al.^149^ applied overcorrection in a group of children (−2.5 D for 12 months, reduced to −1.25 D in the next 6 months), who showed a greater increase in myopia (by −1 D) compared to the control group. Conversely, while in clinical studies, undercorrection has not generally led to a significant decrease in the rate of myopia progression,^150^ a study of the effect of monovision in myopes showed lower rates of myopia progression in the undercorrected eye than in the fully corrected eye.^151^

#### Hyperopic Defocus by Accommodative Lag

Increased lag of accommodation is consistently reported in myopes (see Berntsen et al.^152^ for an extensive literature review). Together with the association between longer and more intense periods of near work in myopes, it can be inferred that myopes are generally more exposed to the hyperopic defocus recognized as a myopiagenic signal. Reports are conflicting whether increased accommodative lag precedes myopia onset^153^ or occurs after myopia onset^83^ (see section 1.3). Additional factors associated with accommodation (crystalline lens, ciliary muscle remodeling, changes in IOP, pupil diameter) and circumstances in which near work occurs (generally indoors) all could play a complementary role to that of hyperopic residual defocus promoting eye elongation.

### Detecting the Sign of Defocus

Similar to the eye's ability to change its refractive power in response to change in vergence even in the absence of proximity cues, it has been argued that the visual system is provided with a mechanism (not necessarily the same as that for accommodation) by which it is able to detect the sign of defocus and modulate axial length to place the image plane at the right focal distance and emmetropize. How the eye detects the sign of defocus and whether this mechanism may be different or compromised in myopes remains a matter of debate. The answers to these questions may add to fundamental understanding of the visual system or even be critical to the design of myopia management strategies. Several cues have been pointed out as potential mechanisms to detect the sign of defocus in emmetropization (an introduction to some of those can be found in Rucker^154^).

#### Spherical Aberration

The intensity distribution of a defocused image in a diffraction-limited optical system for a given magnitude of defocus is identical whether defocus is positive or negative. However, the presence of spherical aberration creates an asymmetry in the through-focus image quality, which has often been hypothesized to possibly disambiguate the sign of defocus.^155^ Thibos et al.^156^ used computational simulations to demonstrate that spherical aberration introduces large differences of contrast between positively or negatively defocused images, with the strength of the contrast difference depending on the magnitude of spherical aberration, the amount of defocus, their relative signs, and pupil diameter (Fig. 2D). These differences are significant over several diopters of defocus, and resistant to the presence of other high order aberrations, for example, those in the periphery. The sign of defocus may be therefore be encoded by a contrast signal.

#### Chromatic Cues

Due to chromatic aberration (see section 1.1), shorter wavelengths in the eye focus in front of longer wavelengths, potentially generating a cue for encoding the sign of defocus. The blue component of the spectrum in better focus than green and red will indicate the eye to elongate (Fig. 2C). On the other hand, a narrowband visual environment could modulate refractive development. Some studies in animal models (chicks and guinea pigs) have shown that a red light rearing environment causes myopic changes (axial elongation, Fig. 2C, lower left inset), whereas blue light causes hyperopic changes, which is consistent with an emmetropization to the best focus at each wavelength, without requiring the chromatic cues present in white light. However, the opposite appears to happen in rhesus monkeys and tree shrews (i.e., a reduction in axial growth and tendency toward hyperopia in long-wavelengths; see references in She et al.^157^ for a review of findings in animal models and Fig. 2D, lower right inset). The latter results in primates have led to the conclusion that proper emmetropization may be impaired in narrowband lighting, possibly because of the absence of defocus-related visual cues provided by LCA (Fig. 2C, lower panel). These cues, based on the differential focus of blue and red, would provide “go” signals for elongation if blue is in better focus than red and, conversely, “stop” signals to slow elongation if red is in better focus than blue,^158^ as illustrated in Figure 2C (upper panel). The Gawne et al. model proposes that the sparsity of S cones in the retina is not an impediment to, in conjunction with longer-wavelength cone mosaics, detecting the differential sharpness in two different planes, as the mechanism would operate at low spatial frequencies.^158^ Furthermore, S cone grating (3 c/deg) detection threshold experiments in emmetropic and myopic humans suggest that myopes may have reduced sensitivity to low spatial frequency S cone stimuli, which might have disrupted normal emmetropization.^159^ A recent study using color (RGB) stimuli that were artificially manipulated to blur the RG components (blue in focus) or GB (red in focus) and presented to subjects at a distance suggested a tendency toward immediate axial elongation (taken as a surrogate of defocus signal detection) when blue was in focus and toward shortening when red was in focus, but only in young emmetropes, not myopes.^160^ Interestingly, Thakur et al.^161^ reported short-term inhibition of axial elongation when young adults with induced hyperopic defocus (−3 D) were exposed to a blue light (but not red or green) environment. The refractive errors of the subjects or time of the day of the experiment were not reported.

#### Central and Peripheral Astigmatism

Along with LCA and spherical aberration, astigmatism has been identified as a potential cue that could guide the initial direction of accommodative change in the fovea, as the combination of positive or negative defocus with astigmatism produces images in different orientations (see Campbell and Westheimer^162^ for one of the first publications in this matter). Recent studies point to the potential role of peripheral astigmatism in detecting the sign of defocus. As described in section 1.1, hyperopes exhibit peripheral myopia and myopes peripheral hyperopia, at least in the nasal visual field. Combined with the fact that astigmatism increases with eccentricity, this results in differences across refractive groups in the orientation of astigmatic blur in the periphery, with emmetropes exhibiting horizontal blur and myopes vertical blur.^163^ The blur orientation in the periphery may therefore encode the sign of defocus. As shown, peripheral cues could combine with chromatic cues, so that the interaction of LCA and oblique astigmatism in the periphery could further result in wavelength-dependent optical anisotropy^164^ (Fig. 2C, upper right inset).

#### Absolute Energy at High Spatial Frequencies

In a theory hypothesized and tested out in a chick model by Hess et al.,^165^ it is the spatial frequency composition of the images, particularly the absolute energy at high spatial frequencies, that would drive ocular growth (Fig. 2E), as the effect is preserved even when the phase of the images was scrambled. This differs from what is generally thought to drive the perception of blur, that is, phase alignments and the relative energy distribution across spatial frequencies, also known as the spectral slope 1/fα. This framework allows separating, for example, a low-contrast and defocused feature in the image and, overall, emphasizes the critical importance of high spatial frequency content in the image to avoid myopia development.^166^ It also may explain why simple undercorrection is not an effective inhibitory signal for myopia.

#### Incremental Retinal Defocus Theory

Hung and Ciuffreda^167^ proposed a theory by which an even-error signal is detected by the retina and encoded by the relative difference in the retinal-defocus magnitude (by form deprivation or directional defocus) during the course of the genetically programmed ocular growth. The proposal considers the different time scales in axial elongation in animal models and human, and it relies on the fact that the myopia onset generally occurs in periods when the eye is still experiencing growth. It also incorporates temporally cumulative effects of decreased retinal image blur produced by near work, which would lead to permanent myopia, upon integrating repeated cycles of, for example, near work–induced transient myopia.

#### Temporal Modulations

In the quest of cues for information about the sign of blur, which cannot be derived from a single snapshot of the retinal image, some investigators have turned their attention to cues grounded in the temporal domain, rather than or in combination with the spatial domain. Earlier work in chicks by Crewther et al.^168^ had shown that emmetropization induced by hyperopic defocus was disrupted under low-frequency (1 to 4 Hz) temporal modulation of the environment during the light cycle, producing an overall myopic shift. This sign-dependent differential disruption of emmetropization could be associated with an alteration of the balance of activation of the ON and OFF pathways. Rather than on the environment, Rucci and Victor^169^ have instead focused on temporal modulations produced intrinsically in the eye by fixational movements. Under this theory, the visual system could estimate the sign of defocus by comparing the amplitudes of temporal modulations, for example, from responses of the retinal ganglion cells. The statistics of the fixational modulations will vary across spatial frequencies, with the amount of blur (Fig. 2G) and also with retinal shape. This information could be used during emmetropization to signal the direction of eye growth. Although differences in eye shape and reported larger fixational error in high myopes^170^ may produce differences in the strength of the temporal cues, whether those precede or are a result of axial elongation remain to be understood.

### Myopia-Triggering Signals

Despite the body of research on potential trigger signals for myopia and potential mechanisms for detecting the sign of defocus, which would drive proper emmetropization, the underlying causes for myopia development in humans remain elusive. If human myopia is comparable to that induced in animals, open-loop (form deprivation) blur or hyperopic blur would be the drivers of axial elongation. While mechanisms for detecting the sign of defocus would probably still need to be invoked, myopia would not necessarily be driven by a failure of those, as the axial elongation response to imposed negative defocus would be a form of emmetropization to increased power (or emmetropization at near, if defocus is created by accommodative lag). However, it is likely that human myopia (slowly developing and probably multifactorial) is more complex than that reflected by animal models where myopia is produced over the course of days with diffusers or lenses.^171^

Attempts to explain the failure to normally emmetropize based on single factors (such as those listed above) face challenges. Many identified potential triggers for myopia, which may stimulate further axial elongation in progressing myopes, have been shown not to precede myopia onset and rather be a consequence of myopia (e.g., retinal shape and peripheral hyperopic defocus). Recent work by Swiatczak and Schaeffel^172^ suggests that emmetropes show a differential short-term growth when exposed to movies with simulated defocus (eye short-term elongation) or seen through positive lenses (eye short-term shortening), but myopic eyes would elongate in either case. Also, in this experiment and in contrast to other theories^165^ (see, e.g., Fig. 2D), the critical frequencies ruling the effect appeared to be relatively low (2 to 8 c/deg).

In similar lines, the chromatic cues that would help to signal the right direction for eye growth appeared to be absent in myopes.^163^ It is not clear whether those observations are a consequence or a cause of myopia and the potential mechanisms behind the differences. If the latter, it is not clear how myopes may acquire specific attributes (low S cone sensitivity, failure to detect the sign of defocus or chromatic focus, higher fixational errors), which may be required by some theories to compromise the detection of the sign of defocus and proper emmetropization. Also, assuming a large role of the environment (rather than predetermined attributes of “to be myopic” eyes), prospective theories should be analyzed under the scope of the spatial, spectral, and temporal characteristics of the environment that subjects are exposed to, including the filtering by their optics.

Some theories for the drivers of myopia are difficult to reconcile. In the classical theory of Wallman and Winawer,^173^ supported by the majority of the evidence presented above, high-contrast images inhibit eye growth, while low retinal image contrast induces axial elongation, and the clinical implication is to avoid scenarios that lead to low-contrast retinal images (Fig. 2D). Conversely, a new “contrast theory”^66^ argues that high-contrast images are in fact drivers for axial elongation (Fig. 2H). Eyes with spurious defective cones through a rare mutation produce abnormally high-contrast signaling, and those tend to be highly myopic. In healthy children, myopia would be driven by high-contrast information, such as written text or the modern visual environment.^60^^–^^66^ In other theories^174^ (Fig. 2I), contrast polarity would differentially trigger axial elongation or inhibition through overestimation of the OFF retinal pathways (black text on white background) and overestimation of the ON retinal pathways (white text on black background). Recent observations from electroretinography studies reveal a deficit in the ON retinal pathways in myopes,^175^ although whether these differences precede myopia has not been investigated.

Finally, to what extent experimental conditions may relate to potentially myopiagenic attributes of the visual environment should be studied with care. For example, while chromatic cues are enticing, does the limited spectral bandwidth required to prospectively produce a failure in the emmetropization mechanism match realistic viewing conditions? Or, in the presence of natural aberrations where the S cone contrast differs little from the M or L cone contrast, would the signal be sufficiently strong to drive the effect?

## Characteristics of the Environment and Myopia

**Figure 3.**
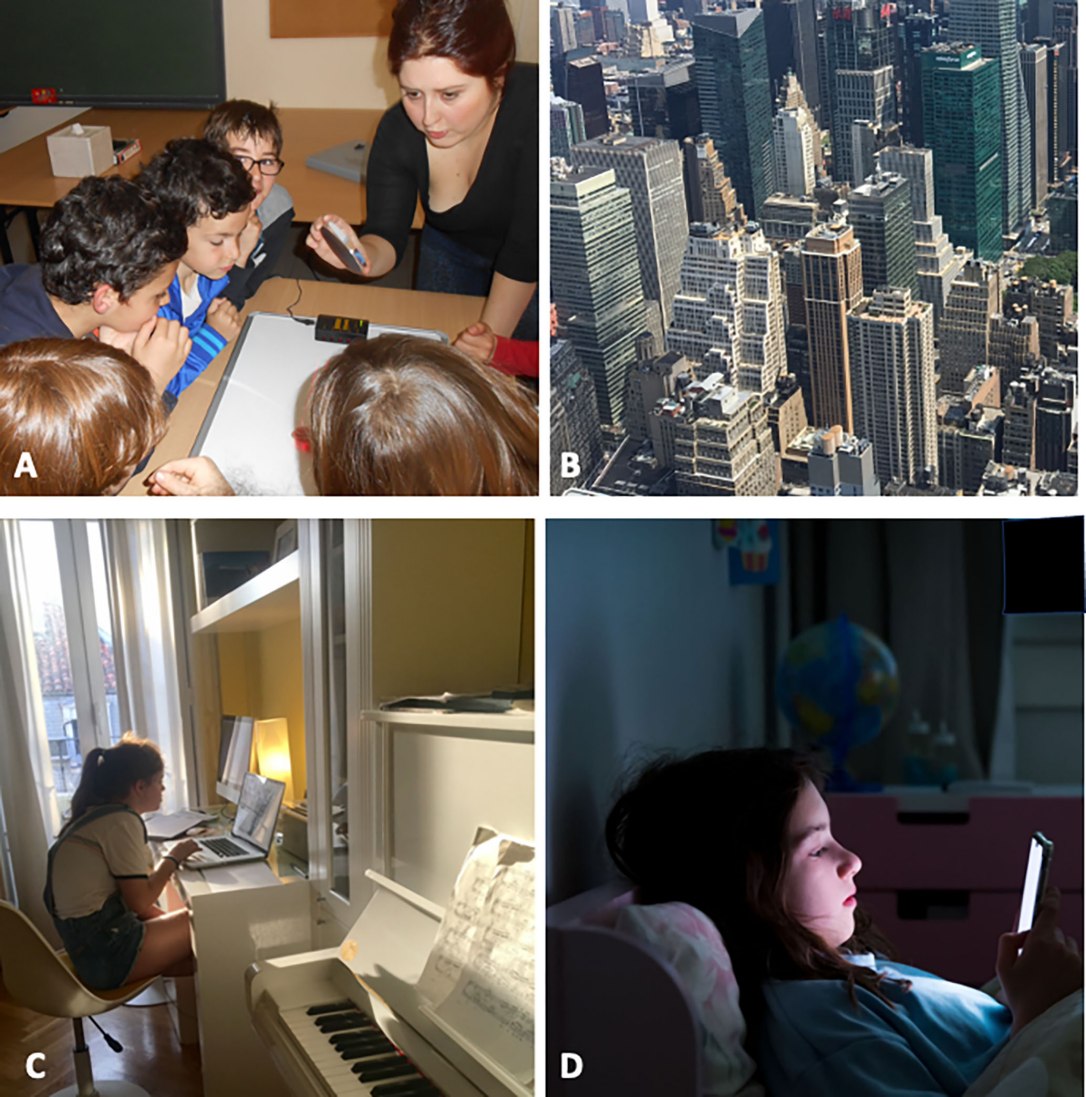
Visual diet and myopia. Examples of visual diet that a child is exposed to during the day, rich in prospectively myopiagenic signals. (**A**) Classroom: indoors, low light, artificial light, near work. (**B**) Urban landscape: reduced spatial frequency range and orientation, steeper spectral slope, higher contrast. (**C**) Homework in the evening: indoors, low light, light with different spectral bandwidth, near work, screen time. (**D**) Digital Displays at night: alteration of circadian rhythm, screen time, near work. (**A–C**) Photography by Susana Marcos. (**D**) Photo from iStock.

The prevalence of myopia worldwide has doubled in three decades.^176^ The largest increase has occurred especially in urban populations of East and Southeast Asia, where 80% to 90% of children are myopic before completing secondary school. These rapid changes, starting to be reported also in other areas of the world, cannot be explained genetically, since gene pools cannot change at those high rates.^177^ A large body of literature is dedicated to the quest of what factors of modern lifestyle can be risk factors for myopia and how those are linked to known triggers of myopia (some discussed in section 2 of this article). Education and near-work activities have been traditionally linked to myopia, and there is an overwhelming association between academic achievement and myopia. More granular studies investigate what specific features of the near-work activity play the strongest role in myopia (reading distance, spatial frequency, contrast, polarity of the stimulus, duration, and intermittency of the activity), as well as the structure of the environment (generally urban settings and indoors) where this activity takes place. Figure 3 shows four expectedly myopiagenic environments. Other studies have focused on illumination, lack of outdoor activities, characteristics of the light, interference of the activities with natural circadian rhythms, and even the latitude, geography, and season of birth. The epidemiological studies are challenging due to close associations between some of the factors under test (e.g., near work generally happens indoors), the need to rely on subjective activity questionnaires, and the lack of control groups. For an extremely comprehensive review of environmental factors and their role in the prevalence of myopia around the world, see Morgan and Rose.^178^

### Spatial Environment and Myopia Development

#### Near Work and Myopia

Near work is considered activities performed at near distance, such as reading, doing homework, writing, playing video games, or using a cell phone. There is an overwhelming amount of literature associating near work with myopia. A review and meta-analysis by Huang et al.^179^ based on 27 questionnaire-based studies on >25,000 children from Asia, North America, Australia, Europe, and the Middle East concludes that individuals who perform more near-work activities (particularly reading) have an 80% higher risk of having myopia. A recent systematic review by Gajjar and Ostrin^180^ also points to relations between myopia and near work from longitudinal studies and emphasizes the working distance (<30 cm) and duration of uninterrupted near-work activity (>30 minutes) as risk factors for myopia onset and progression. Large epidemiological studies are very consistent in showing associations between myopia and academic performance in populations with a similar ethnic background and geographical locations. For example, in a cross-sectional study on >32,000 Chinese school children from large cities in Guandong, China, the prevalence of hyperopia was associated with lower academic scores in grade 1, and the prevalence of myopia was associated with higher academic scores in grades 6 and 8.^181^ Longitudinal studies showed that faster myopia development was associated with higher academic scores in all grades (also after adjustment for body mass, outdoor activity time, and parental myopia).^181^ A paradigmatic study supporting the impact of near work on the prevalence and severity of myopia comes from a comparison of educational systems (>22,000 male adolescents from ultra-Orthodox, Orthodox, and secular) in Israel.^182^ The prevalence of myopia in the ultra-Orthodox schools, which involve intensive reading since early childhood, a high number of study hours, and a very close viewing distance, was 82.2%. This prevalence is much higher than 50.3% in Orthodox schools and 29.7% in secular schools.

Which aspects of near work represent risk factors for myopia development, which metrics should be used to define near work, and which features of the retinal image in the accommodating eye are the most relevant questions in addressing the “visual diet” of developing myopes. The following two main factors (see Morgan and Rose^177^ for references) are identified: viewing distance (accommodative lags increased with decreasing vergence) and interruptions to the near-work activity (intermittent periods of myopic defocus have been shown to counteract the myopiagenic action of hyperopic blur). In addition, the accommodative response depends on the spatial frequency content of the image (peaking at mid spatial frequencies), stimulus contrast, and retinal illuminance, and these stimulus factors will expectedly determine the residual defocus on the retinal image.^183^^,^^184^ Other theories that relate near work with myopia development through other mechanisms include the lower-contrast retinal image conditions arising from the combination of residual accommodation and negative spherical aberration^156^ associated with accommodation (Fig. 2D) and overstimulation of OFF retinal pathways by black text over white background^174^ (Fig. 2I).

#### Urban Versus Rural Environments

Morgan and Rose^178^ reviewed dozens of epidemiological studies around the world, sorting them between those conducted in rural and urban environments. Consistently, the prevalence of myopia is higher in urban settings and lower in rural settings.^185^ Several studies have investigated differences in myopia prevalence and progression in urban and rural individuals with similar ethnic backgrounds.^186^ It is likely that a large part of the observed differences in refractive errors arises from differences in educational pressures and socioeconomic indices, which tend to be higher in urban compared to rural environments. Children in rural environments are likely more consistently exposed to long-distance viewing and spend more time outdoors. However, other direct myopiagenic effects of growing in a built-up environment may be considered. Flitcroft et al.^187^ have demonstrated that urban and indoor environments exhibit different spatial properties than natural outdoor environments. Humanmade urban and indoor environments have a lower high spatial frequency content with a steeper log spectral amplitude versus log spatial frequency (slope: −1.50) compared to natural environments (slope: −1) (Fig. 2F). The authors argue that the deficiency of high spatial frequencies in artificial environments could mimic those produce by diffusers. If the retina simply estimates defocus from the slope of the amplitude-spatial frequency in the image at, for example, a middle-frequency range or by analyzing the energy at high spatial frequencies, then the analysis of the artificial images would trigger an eye growth signal, as a blurred image would, and therefore impact myopia.

### Ambient Light and Myopia Development

#### Outdoor Light Exposure

Numerous studies, reviews, and meta-analyses discuss the impact of outdoor exposure on myopia development and myopia prevention.^188^^–^^192^ In general, children who spend more time outdoors exposed to natural light tend to be less myopic than those who spend less time outdoors. Debate still exists whether increased time outdoors slows down myopia progression in preexisting myopes. As with other investigated factors, more research is required to investigate causality and to determine which properties of the outdoor lighting impact myopia and the mechanisms behind light-induced (or deprived) myopia development. The illuminance levels (amount of visible light per square meter corrected by the spectral sensitivity in humans, measured in lux) differ greatly between indoors (100 to 500 lux) and outdoors (where the sun can exceed 100,000 lux). Norton and Siegwart^193^ proposed a model that superimposed normal refractive development to periods of accelerated axial growth in response to myopiagenic stimuli at low light levels and a protection from myopia development at elevated light levels, which would activate retinal dopaminergic pathways controlled by dopamine amacrine cells (contributing to the retina's ON and OFF pathways) and inhibit axial elongation.^194^

#### Light Properties (Timing and Wavelength), Circadian Rhythms, and Myopia

Evidence coming primarily from animal work (see, e.g., a review by Nickla^195^) shows that abolishing diurnal cues (i.e., constant darkness or constant light) disrupts normal emmetropization. On the other hand, studies in different animal species have shown alterations in the diurnal rhythms of axial elongation and antiphase choroidal elongation in response to form deprivation and negative lens rearing.^196^ Chakraborty et al.^197^ have proposed pathways that incorporate the internal retinal clock and eye growth rhythms in the regulation of postnatal eye growth and emmetropization. Light (and possibly blur) properties of the visual images projected on the retina interact reciprocally with the retinal circadian clock, synchronize it to the diurnal cycle, or desynchronize it under disrupted physiological conditions. Additional support to the role of circadian rhythms in emmetropization comes, among others, from observations of the involvement of retinal melanopsin signaling in emmetropization in murine models.^198^^,^^199^ Animals with disrupted melanopsin retinal ganglion cells showed alterations in the rate and magnitude of refractive development, susceptibility to form deprivation myopia, and modifications of dopamine signaling.

In modern times, the availability of artificial light has substantially changed the light environment. Indoor schooling reduces the benefits of sunlight exposure during the day (Fig. 3A), but artificial light has prolonged light exposure during evening and night hours (Figs. 3B, 3C). Melatonin suppression and shifts in the circadian rhythms are modulated by the amount of light seen during the day as well as the temporal and spectral content of the light. In general, high light intensity levels during the day favor alignment of endogenous circadian rhythms and external light–dark cycles. On the other hand, blue-rich LED artificial light and computer and phone displays^200^ in the evening alter circadian rhythms and sleep in humans, as well as the physiological processes involved, including melatonin suppression.^201^ Interestingly, a recent study by Chakraborty et al.^202^ found altered circadian timing, lower melatonin, and sleep disruption in myopic children, supporting the interactions between circadian rhythms and myopia development, although what is cause and consequence remains unclear. Not surprisingly, several studies have found strong associations between smartphone use and myopia (see, e.g., Mccrann et al.^203^ and Foreman et al.^204^ for a review and meta-analysis), where near work, and likely use during the evening, may reinforce myopiagenic signals. As noted by Stone et al.^205^ in a recent report, the likely involvement of circadian rhythms in myopia development makes it critical to emphasize the time of day of mechanistic studies of myopia.

### Interactions Between Environmental Factors

Most of the factors described in sections 3.1 and 3.2 are largely interwoven. Near-task activities are most often performed indoors, and the types of activities performed outdoors generally entail less near-vision pressure. Given the codependency of many of these factors, it is difficult to attribute the risks of developing myopia to one specific factor.

For example, seasonal variations in eye growth have been reported in several studies (see, e.g., Gwiazda et al.^206^), with a deceleration of myopia progression in the summer and acceleration in the winter. But is this associated with the sunlight hours or the seasonal differences in school attendance and homework? The potential association of circadian rhythms in myopia development has caused some investigators to look at latitudinal variations in light intensity and diurnal durations across the world. The report of a higher prevalence of myopia in adults born in the summer months (reported, for example, in the United Kingdom and Israel) is probably unrelated to visual factors.^207^ On the other hand, reports of a relatively low prevalence of myopia in children in Scandinavian countries,^208^ with less hours of sunlight, particularly during the winter months, may be rather related to a higher emphasis on spending time outdoors, for example, in Norway, where outdoor time during recess is the norm, regardless of the time of the year.^209^ In China, the introduction of 40 minutes of outdoor class activities added each day at schools appears to have had positive outcomes in reducing the incident rate of myopia.^210^ Recent studies report increased myopia progression and incidence following the lockdowns imposed by COVID-19, which drastically reduced time outdoors and increased screen time.^211^ Studying associations between myopia progression and the severity of the home confinement, type of housing, and near-work activity during pandemics could give further insights into the interactions of those factors in relatively more controlled settings.

The increase in myopia prevalence has preceded the digital age and can be highly prevalent in populations with limited access to smartphones.^182^ However, it is very likely that the factors discussed above have a cumulative effect,^212^ and they all contribute to increasing the risk of myopia and its development, perhaps through nonlinear interactions and triggering multiple pathways. Leaving genetic susceptibility and parental myopia aside, a child who spends a long time performing near work indoors with the targets too close and poor illumination will likely be exposed to blurred images by the presence of accommodative lag and will be largely deprived of the benefits of outdoor daylight exposure (including dopamine release). Living in an urban environment, the spatial frequencies will be reduced not only in the indoor settings but also outdoors, limiting the benefit of the distance viewing breaks (Fig. 3B). Using artificial lighting while doing homework at home at night (Fig. 3C) and prolonged smartphone use (Fig. 3D) will not only increase the near-activity hour count but also potentially disrupt circadian rhythms and melatonin secretion, further contributing to axial elongation.

### Technologies to Assess the “Visual Diet”

The intricate connections between lifestyle and environmental factors make it difficult to segregate their individual effects. This complexity underscores the need for studies that use objective assessments of light exposure and near work, among others. Increasing understanding of how various environmental factors affect myopia development is critical and prospectively will give hints about how they can be modified to prevent or slow the progression of myopia.^213^

Many reports of lifestyle and outdoors exposure in relation to myopia are based on questionnaires, generally to parents, and those have proven to be noisy and inaccurate. In particular, a comparative study has shown that the self-reported questionnaires on visual activity overestimate objective measures obtained from the Rangelife, a device monitoring working distance.^214^ There is an emphasis on replacing the questionnaires with objective tools to assess near work and the physical properties of the space.^215^

To build a model of the luminance, spatial, temporal, and chromatic environments, real-time monitoring of outdoor exposure from the point of view of the observer’s eye is needed. There are published reports of the use of light meters, accelerometers, and range finders mounted on spectacles, as well as recent reviews of different approaches for measuring illumination and distances.^216^^,^^217^ The systems come in different formats and are attached to different parts of the body. For example, Harb et al.^216^ presented a study using a wrist-mounted device (Actiwatch). Some devices (i.e., a custom watch connected to a smartphone, FitSight Fitness tracker) not only measure activity but also aim at inducing behavioral changes according to the recorded activity.^218^ Other commercial devices, reviewed in the above references, include watches (Actigraph) or cloth-mounted portable sensors (HOBO) to record illuminance.^216^^–^^219^

It is recognized that the most valid device should make recordings at the eye's line of sight to capture the light and spatial content that reaches the eyes, rather than just ambient light. Gibaldi et al.^217^ presented a bicycle helmet–mounted device measuring intensity, distances, and spectral content in the environment. Wen et al.^219^ reported on the Clouclip, a wearable device that measures working distance and eye-level illumination, integrating data captured during daily wear for 1 week.

## Supplementary Material

Supplement 1
