# Supplementary material for: Optical and Visual Diet in Myopia
Source: Invest Ophthalmol Vis Sci. 2025 Jun 5;66(7):3. doi: 10.1167/iovs.66.7.3 (PMC12151263; doi:10.1167/iovs.66.7.3)
Supplement: Supplement 1 [file iovs-66-7-3_s001.pdf]

ANNEX. OPEN QUESTIONS

Three questionnaires were prepared by Prof. Susana Marcos and distributed to experts in the field of myopia, vision and visual optics. The questions aim at debating open questions and potential directions of research. The authors and their responses are transcribed below.

QUESTIONNAIRE 1. OPTICS OF THE MYOPIC EYE

Prof. David Atchison	School of Optometry<br>Queensland University of Technology, Australia<br>d.atchison@qut.edu.au	**DA**
Dr. Stacey Choi	School of Optometry<br>Ohio State University, USA<br>choi.1080@osu.edu	**SC**
Prof. Michael Collins	School of Optometry<br>Queensland University of Technology, Australia<br>m.collins@qut.edu.au	**MC**
Prof. Rhea Eskew	Department of Psychology<br>Northeastern University, USA<br>r.eskew@northeastern.edu	**RE**
Dr. Arthur Ho	Brien Holden Vision Institute and The University of New South Wales, Australia<br>a.ho@unsw.edu.au	**AH**
Prof. Fabrice Manns	Bascom Palmer Eye Institute; Dep. Biomedical Eng. University of Miami, USA<br>fmanns@miami.edu	**FM**
Dr. Jos Rozema	Department of Ophthalmology<br>University of Antwerp, Belgium<br>jos.rozema@uantwerp.be	**JR**
Prof. Austin Roorda	Herbert Wertheim School of Optometry and Vision Science, University of California, Berkeley, USA<br>aroorda@berkeley.edu	**AR**
Prof. Maciej Wojtowski	International Center for Translational Eye Research , Poland<br>mwojtkowski@ichf.edu.pl	**MW**

1.- The ***crystalline lens*** *of the eye has probably not received the attention it merits as a agent in myopia development*

* What is in your view the role of the crystalline lens in emmetropization and myopia development?

* Would longitudinal studies help to determine if the lens has an active or passive role in myopia development?

JR: Q1: The crystalline lens plays a major role in both emmetropization and myopization as it is the only optical structure that can compensate for excessive axial growth. This is clearly seen in fast growing eyes that are not yet myopic, where the lens power loss occurs faster than in eyes with a normal growth rate. Once the lens reaches a certain minimum value below which it can no longer lose power efficiently the eye as a whole will become myopic. This in turn will gradually slow the

axial growth). In my opinion the lens power loss is mostly a mitigating response to the rapid axial growth, but it is conceivable that the lens also has a minor influence on the axial growth rate. Regardless, most clinicians do not seem to consider crystalline lens power as important, which is reflected in the fact that it is rarely included in myopia studies and there are no clinical devices that provide this value. Adding a lens power estimate to the screen of common biometers (e.g., Lenstar or IOL Master) would help address this knowledge gap.

Q2: There have already been several longitudinal biometry studies that looked into lens power changes (see e.g., the references above) and several others that can be repurposed to calculate lens power. Deeper analysis of those studies can teach us more, but new studies with better equipment are always welcome. One important point of interest would be the influence of myopia control solutions (spectacles, contact lenses, atropine…) on the lens power. One of our recent analyses showed that atropine 0.01% allows the crystalline lens to lose power again for a while after the onset of myopia, thus reducing progression. More observations could therefore teach us about the mechanisms underlying myopia and myopia control.

MC: The crystalline lens appears likely to be an active contributor to the process of emmetropization, becoming thinner and losing power synchronously as the eye grows longer in childhood. I think longitudinal studies of the age-related changes in the crystalline lens in emmetropic, myopic and children becoming myopic would be extremely valuable. The more information we can have about the changes in the lens and the related biometry and optical changes, the greater our chances of determining whether the changes are passive or actively involved in myopia development. A study of this type would benefit from detailed measurements of the ciliary muscle, OCT measurements of lens morphology and thickness, estimates of refractive index changes and wavefronts generated by the lens. I think a key component of this study should include the measurement of these lens and lens-related features across a broad range of accommodation levels. We tend to think of the eye as a static system focused into the distance, but it is dynamic and the eyes of children spend the vast majority of time focused on objects close to their eyes. Acquiring these measurements with active accommodation is challenging, but achievable and necessary.

AH: While only speculative given our current knowledge, there is a suspicion that the lens is relevant to emmetropization and/or myopigenesis. There are 'coincidences' in the growth rate of the lens (e.g. age range at certain inflexion points of lens growth is also the typical age range of childhood myopia onset). The long time-course and wide individual variations in emmetropization/myopigenesis necessitate longitudinal studies for investigations to be valid - especially in understanding whether the lens plays a passive or active part.

RE: I do not know the role of the lens. But It seems likely that a longitudinal study of its role would be informative.

FM: Q1 In my view the lens plays a critical role but most likely a passive role. The lens remodels significantly as the eye grows during the phase of emmetropization and myopia onset. This remodeling occurs faster than can be explained by the intrinsic growth of the lens (the addition of new lens proteins). Lens remodeling occurs at much faster rate than can be explained by intrinsic change in lens volume. This suggest that lens remodeling is a passive mechanism due to external forces that increase the tension on the zonules. Zonular tension may increase due to increased zonular tension as the outer shape of the globe changes during ocular growth. It could be due to equatorial growth or maybe more likely to axial growth which moves the posterior zonular attachment posteriorily and increases zonular tension. Independent of the mechanism, these

changes produce changes in lens power and aberrations across the visual field which work with ocular elongation to determine reitnal image quality.

Q2. Yes. There have been longitudinal studies, but they have been limited to correlating lens or thickness with axial length and refractive error. To help truly quantify the role of the lens, longitudinal studies should include the entire ocular biometry, lens diameter, peripheral optics and accommodation.

*2.- Building **computational myopic eye models** is of interest to predict the interactions of the eye's optics to the optics of correcting lenses, while age-dependent models would allow to evaluate the association between geometrical and optical changes with normal emmetropization, myopia development and myopia control. The eye models rely on accurate structural information of the eye, and can be informed by aberrometric measurement.*

* How can eye models based on population data will help advance the field?

* Will large intersubject variability in geometrical parameters be a limitation?

* What are current technical limitations of standard biometry techniques to generate reliable eye models (on axis and off-axis)? Where efforts should go in biometry that will help the field?

* How biometry and aberrometry can assess whether biometry and optical changes are a cause or consequence of myopia development?

DA: We do not know the refractive indices well enough to obtain accurate thicknesses of components in the eye, nor how refractive index varies across the lens; most if not all instruments do not obtain vitreous depth directly, but rely on the difference between axial length determination based on a "fits all" refractive index and the sum of other component lengths The last [biometry/optical change] cause or consequence requires longitudinal studies to determine the relationship between the changes in these parameters and the changes in refraction, e,g, if one group of people develops myopia and another group doesn't, are parameters different before or while the refraction of the groups is diverging.

JR: Q1+2: There is a large variation in the ocular biometry of individuals and there are many ways in which an eye can be emmetropic or ametropic. For example, emmetropes can have axial lengths between 21 - 26 mm. At the same time this range of axial lengths sees refractive errors between -8.5D and +6D, illustrating the large overlap between the biometry of the different refractive groups. This makes it difficult to define clear boundaries between refractive groups based on biometry alone. One issue is that emmetropia is still rather poorly understood as a concept. Emmetropia can only exist if the different optical components are correlated with the axial length and each other. Hence, it is important to study the refractive development during infancy and childhood that establish these correlations over time (emmetropization) and preserve them in the long term (homeostasis). Meanwhile, deviations in this refractive development will often lead to myopia. Longitudinal population data are therefore essential to create mathematical models of normal and abnormal ocular growth that consider the correlations between the components. Ideally these models start at the earliest possible age, some time before the children go to school, so the existing growth before the myopogenic influences is included as well.

Q3: I see three limitations: (1) Apart from lens thickness, lens biometry is still not widely in the clinic. Any method that could simultaneously provide accurate estimates of the corneal and lenticular curvatures and thicknesses, and perhaps even the lens gradient index profile, might be

useful. Ideally, this would come in the form of a portable system that could be taken to schools for population studies in children. (2) Many clinical devices were not developed with children in mind, so their calibration might be a bit inappropriate for the eyes of young children. Moreover, young children have more trouble fixating on a target for the duration of a measurement, reducing the accuracy. (3) It is difficult to perform off-axis biometry measurements in a clinical setting to follow-up peripheral changes. One could imagine a wide field biometer, similar to the VOptica scanning aberrometer, that could create instant wide field models of an eye to address this.

Q4: This is a difficult question to answer. Biometry and refractive error are linked in a closed retinal feedback loop, where axial growth responds to the refractive error, which in turn affects the refractive error. Before industrialization myopia was not very prevalent, and it is safe to assume that since then no major changes took place in scleral stiffness or the retinal response in the general population. So if the feedback mechanism has not changed since that time, myopia is likely results from changes in the way eyes are being used (more near work, more work in the dark or in artificial light, less time spent outside). Some people may also be more susceptible to these impulses because of variations in scleral stiffness or retinal response. So, biometry may be a cause (or rather risk factor) for myopization, while at the same time myopization will have major consequences for biometry. So both statements are true. To determine whether an eye is at risk of becoming myopic in practice, most clinicians look at the current biometry (typically refractive error and perhaps axial length). But as myopization is a process that starts some time before the eye becomes myopic, it is far better to consider biometric changes over time, while also considering the correlations between the biometric parameters. But to do this right, children should be screened regularly to identify those at risk of developing myopia. As this would be very costly, it would be interesting to link this screening to additional risk factors, such as hereditary, behavioral, and environmental factors. Ideally, one would link this to physiological low scleral stiffness or a slow retinal response, but to date there are no clinical devices available for this purpose.

AR: Q1 Models: I completely agree that we need better eye models. In fact, the Center for Innovation in Vision and Optics at Berkeley hired a postdoctoral fellow to develop a wide-field eye model. We'll be publishing the details soon. We had several aims for the eye model: (1) To better model the optical quality in the periphery. It is becoming clearer that peripheral image quality might be a strong factor governing eye growth or driving myopic and multiple interventions that aim to slow the progression of myopia are aiming to control peripheral image quality. (2) To enable proper optical evaluation of interventions that aim to slow progression of myopia. Purveyors of some optical interventions purport that their treatment manipulates the defocus in the periphery. But where in the periphery? Does the defocus change also come with a change in astigmatism? Or high order aberrations? How do the optical effects of these devices changes with pupil size? The actual effects of these optical interventions can be predicted with a proper eye model. Importantly, if indeed the mechanism of action of these lenses are understood, then the model can be used to optimize their designs.

Q2 Intersubject variability: This is definitely important. A single eye model is likely inappropriate. I think that, at a minimum, there should be different eye models for different populations. Having separate eye models for emmetropes and myopes is a start. Our plan is to release eye models for multiple eyes in each category which will help to capture the interindividual variation.

Q3 Biometry: The more biometry the better. While the overall wavefront (and therefore image quality) can be computed fairly accurately, the specific details of the optical system that generates the wavefront cannot. I always tell my students that no one in the history of the world has ever measure biometry of the eye well enough to predict the image quality. Susana Marcos is perhaps

the closest to have done that, but she has been most successful for eyes with implanted intraocular lenses (simpler for sure, but still a very impressive feat). I think that, moving forward, OCT-based biometers will be a key technology to tackle this problem.
Q4 Cause or consequence? If you believe that myopia progression is primarily through a simple lengthening of the eye, then it is possible that aberrometry and biometry might still reveal the original optical factors that give rise to the eye growth even after it has taken place. But I suspect that it will not be that simple.

MC: I think the key issue is to have longitudinal studies that allow us to establish whether the biometric and optical changes occurring in the children's eyes occur before or after myopia development. Cross-sectional studies can't answer this question with confidence.

AH: We have known for decades that eye-growth can be (among other factors) optically guided. More recent studies suggest that optical characteristics beyond 'mere' defocus may be influential - such as retinal position of the optical effect, contrast, colour/wavelength, brightness of the lighting environment, etc. Yet, there is no firm understanding of the underlying mechanism(s) involved with the various optical interventions. Clinical studies of myopia development, including efficacy of treatment methods typical measures only gross aspects of refraction such as sphero-cyl components. To gain insight into how visual optics drive emmetropization, a much more refined approach is needed - such as understanding the role of ocular aberrations, their effect on retinal image quality (e.g. PSF). A computational eye model for studying refractive error (not just myopia, but hyperopia - as the infant eye is typically hyperopic) incorporating such parameters as aberrations, geometry, etc, can provide valuable insight into the optical characteristics that may drive emmetropization (or stimulate myopia). While some eye models already incorporate refractive error (e.g. Atchison 2006), an eye model that is directed to understanding myopigenesis should ultimately be far broader in scope. In addition to computing, e.g. point spread across the retina from ocular aberrations, since neural-retinal factors have also been suspected to be relevant, a holistic eye model may need to incorporate the neural-physiological response beyond the image on the retina (e.g. Timar-Fulep, 2019). These may also include SCE (both kinds), S/M/L cone responses, receptor fields, etc. My view is that it is the intersubject variability, and our ability to correlate their effects on eye growth response that will provide the greatest in-depth knowledge into refractive error development. For this reason, and to meet this purpose, customised eye models of refractive error development that can be configured to match individual biometric and optic data should be a major objective. From the ocular biometry standpoint, in general, the accuracy of most measurement systems needs improvement to be commensurate with the level of optic computations that may be needed in this field. However, one area that particularly needs development is in wide-field measurements. It is known that peripheral optics of the eye can direct refractive development. Techniques to measure reliably peripheral geometry and optics of the whole eye and the spatial position of the ocular components relative to each other needs further development. In particular, measurement systems for the crystalline lens, due to its gradient index and ability to accommodate also needs further development.

RE: Eye models that allow for individual difference adjustments are crucial, and not only for myopia. They can help understand (and treat) changes with age, for example. Separating cause and consequence is a second-order effort and these models can only provide a start on such questions.

FM: Q1. Model eyes base on population data can help by enabling computational studies to evaluate the contribution of individual parameters (distances, curvatures, pupil) on the retinal image quality. This can help identify which parameters have more weight on determining retinal image quality and can help simulate the rate of change of the retinal image in terms of the rate of

change of individual parameters. These models will also help assess optical corrections, for instance the role of continued ocular growth or accommodation on the retinal image.
Q2. Yes. In my view, we need both population-based models and individualized eye models. There is significant interindividual variability of both the anatomical parameters and the growth rate a given age. A model based on a population average will not capture these variations, and a simple statistical distribution of individual parameters will also not capture this variability. It will be important to have models that capture the intercorrelation between anatomical the various anatomical parameters.
Q3. Current commercial biometry devices do not provide accurate measurements of the whole eye (cornea + lens + retinal shape) and do not simultaneously measure ocular anatomy and peripheral refraction. They are also not easily setup to study the accommodative response. In my view it will be important to develop standardized protocols for ocular biometry with commercial devices so that results can be compared across studies, including rigorous quantification of measurement precision and repeatability, and to include the individual measurements in publications instead of just averages. It is also important to develop instruments that can easily measure refraction, ocular distances, and aberrations across the visual field (center and periphery). Many many studies find a large interindividual variability particularly in lens biometry (thickness, power and curvature) and in the correlations between refraction and biometry. Generally there is no rigorous measure of repeatability, so it is unclear how much of the interindividual variability is intrinsic, or due to limited measurement precision. In addition, many of the studies on lens power rely on an approximate method (the Bennett method), which introduces a significant source of error when applied to young eyes. given that changes in lens power are generally small (0.5 to 0.7D/year), it is critical to develop more precise techniques to measure of the lens shape and power, or at least carefully assess the measurement error in these studies. MRI is a great modality, but the cost, logistics and motion artifacts limit its applicability for large scale longitudinal studies.
Q4. That's a tough "who comes first: chicken or egg?" question. This is where the combination of longitudinal studies and anatomically-accurate computational eye models are important, and also perhaps longitudinal studies in eyes who receive optical treatments or studies on animal models. Perhaps one way to determine the cause-effect relation is to develop two closed-loop control systems corresponding to each of the two hypotheses and compare the model predictions with the results of longitudinal studies. Comparing the dynamics of the retinal image predicted from biometry using computational eye models with the measured retinal image from aberrometry we should be able to determine causality. Perhaps it not one or the other but a combination of growth modulated by optics and vice-versa (i.e., a closed-loop control system).

*3.- The role of **accommodation** in myopia has been a matter of debate.*

* Can we say that myopes are lousier accommodators?

* Has the study of accommodation in myopes been hampered by the limitations of existing methods to measure the accommodative lag?

* How should accommodation be properly measured? Will that technique allow to measure accommodation through multizone lenses?

DA: I think that there is enough evidence for the answer to the first question to be yes, but the accuracy of accommodation measurements has been called into question. I think the objective methods of determining aberration coefficients are good, but the issue is how these coefficients are used to determine appropriate refraction/accommodation. As far as I aware, the aberration must be determined through a path that includes only one power e.g. in a concentric bifocal, only

one zone must determine the response. There is a paper by Marty Banks' group (Journal of Vision) that claims that accommodation is much more accurate than previously believed, but there were only a few subjects and no emmetropic/myopic comparison.

JR: Q1: I'm not sure if myopes must have poorer accommodation to become myopic, but it can play an aggravating role. There have been a few studies suggesting an accommodative lag in myopes that would support this.

Q2: Many reports studied accommodation in one eye by stimulating the fellow eye, which does not seem optimal. There is also a lack of uniformity in reporting (e.g., some papers report stimulus, others report the response), so any effort to harmonize reporting would be beneficial. Q3: OCT or Scheimpflug images with appropriate correction for optical distortions should work well for the physiological situation, but may experience some issues with multizone lenses

AR: Q1 Lousy accommodators? In my opinion, the jury is still out on this. We first need better ways to measure and elicit accommodation before we can say anything conclusive.

Q2 Limitations: An artifact of so-called 'accommodative lag' could arise because of technical limitations of the optical measurement itself, or a failure to set up proper conditions to elicit accommodation in the first place. In the natural world, many monocular and binocular cues are present to help guide accommodation. If these are not all present during the measurement, then we should not assume that they will be accommodating properly. We need better ways to measure accommodation.

Q3 Measuring accommodation? First, we need to agree on a definition of accommodation. Accommodation may be computed as a physical change in the optics that gives rise to a change in the axial distance to an object that casts that sharpest image in the retina (often converted to a change in diopters of vergence from that object). Given that definition, then we need to agree on what is the 'sharpest' image, which in itself is a metric that is criteria dependent. But researchers are learning what factors govern perceived sharpness (e.g. visual Strehl ratio) and with modern wavefront sensing technology, we are able to compute image quality, not just at the retina, but at all axial distances. I think it is just a matter time before all this knowledge coalesces into a device that will provide very reliable estimate of accommodation. We've taken some steps in that direction in a recent paper. Roorda, A., Tiruveedhula, P., Naseri, A., Rhee, P. and Clarke, M., 2023. FIAT: A Device for Objective, Optical Measures of Accommodation in Phakic and Pseudophakic Eyes. Translational Vision Science & Technology, 12(1), pp.9-9.

A bit more about accommodation… I think we need to consider the notion that some of the factors that drive accommodation - which are essentially reflexive and involuntary - might overlap with factors that guide eye growth. For example, my colleagues show a paradoxical result where they manipulated chromatic cues on 2D rendering in a way that caused the eye to accommodate away from the screen, even though it made everything blurrier! Cholewiak, S.A., Love, G.D., Srinivasan, P.P., Ng, R. and Banks, M.S., 2017. Chromablur: Rendering chromatic eye aberration improves accommodation and realism. ACM Transactions on Graphics (TOG), 36(6), pp.1-12. If such chromatic signals can so effectively drive accommodation, then why would they not be leveraged for growth (or to cause properly controlled eye growth to go off the rails)?

MC: I don't think myopes have worse accommodation. If they were poor accommodators, their visual resolution would be measurably worse than emmetropes and it isn't. There are differences in higher order aberrations between young myopes and emmetropes during accommodation that

can make it appear that their accommodation is worse. Most autorefractors “average or sample” the optics of the eye in various ways and that produces misleading results because of the higher order aberrations of the eye. Wavefront sensors have sufficient resolution for the job, but the open question is what is the best method to analyse the wavefront to best represent the state of “accommodation”? Measuring “through” contact or spectacle lenses is tricky. You can measure the wavefront of the eye without the lens in place and then with the lens in place (eg both with cycloplegia). The difference in wavefronts is then the effect of the lens optics, independently from accommodation. But factors can change the optics such as contact lenses moving on the eye with blinking and not returning exactly to the same location. The other factor is the downward gaze and convergence associated with accommodation during near tasks. These eye movements affect contact lens centration and line of sight through the spectacle lens and possibly also affect the shape of the eye and the biomechanics of accommodation. One way to get the accommodation response to multifocal optics is to create the multifocal optics in an adaptive optics vision simulator and simultaneously measure the change in “accommodation” with a wavefront sensor. If eye tracking is combined with the system, the multifocal optics can be centred or decentred by fixed amounts using real-time feedback to the adaptive optics system. But these optical systems typically don’t simultaneously allow manipulation of convergence and downward gaze, so these biomechanical factors are not included in the measurements.

AH: Literature on the relevance of accommodation (e.g. lead/lag) on myopia appears to be equivocal. One possible confounder is that such studies typically measure the steady-states of lead/lag. It may be that the dynamics (speed of response, stability of response at end-points) of accommodation is relevant. Another is that studies are necessarily undertaken in relatively artificial conditions whereas 'real world' accommodative responses may vary according to the visual environment.

RE: I believe the literature, taken as a whole, indicates that myopes are worse at accommodation. Better dynamic techniques for measuring the lag would be very helpful.

FM: Q1 Due to increased eye length, myopes need less of a lens power change to produce the same accommodative effect. In addition at fixed pupil diameter the numerical aperture in the image space (retinal) is smaller in myopes, which should provide more depth of focus. So in my view, myopes are optically-more-efficient accommodators, rather than more lousy accommodators.

Q2. In general, there are not that many studies comparing the full accommodative response in myopes and emmetropes. Most of these studies is that they rely on static measurements of accommodative response at a few discrete accommodative demands. In our own experience, we find significant interindividual variability in accommodative responses when measured at discrete accommodative steps, even among emmetropes. I believe that a continuous dynamic measurement of the accommodative response to a ramp stimulus or perhaps sinusoidal stimuli would help more accurately capture and isolate the effects of lag and response.

Q3. In my view, a dynamic measurement of accommodative response to a ramp stimulus under binocular conditions with a wavefront aberrometer or some other dynamic objective refraction method (e.g., photorefraction) that provides a continuous measurement. Monocular stimuli may also be satisfactory depending on the goals of the study. Measurements through multizone lenses could be performed with a laser-ray-tracing approach at fixed or variable optical zone or a different type of zonal approach.

*.4.- Adaptive Optics retinal imaging has allowed quantifying **cone spacing** in and outside of the fovea, and the differences between myopes and emmetropes have been investigated.*

* How can additional studies of retinal cone spacing shed light into the anatomical changes undergone by the eye as myopia develops?

* Could differences in observers cone mosaics (in particular S-cone mosaics) in the fovea and parafovea play a role in the detection of emmetropization (or myopia triggering) signals?

AR: Q1 Cone Spacing: We have shown that lengthening of the eye will cause in increase in angular retinal sampling by the cone mosaic. Wang, Y., Bensaid, N., Tiruveedhula, P., Ma, J., Ravikumar, S. and Roorda, A., 2019. Human foveal cone photoreceptor topography and its dependence on eye length. Elife, 8, p.e47148. But there is still a lot to learn. For example: High resolution imaging might reveal information about the physical stresses that excessive eye growth puts on the photoreceptors and supporting cells, like the RPE and inner retinal neurons. Also, the combinations of imaging and vision testing might reveal important structure-function relationships of the photoreceptor cells and downstream neurons that results from eye growth or that may trigger excessive eye growth.

Q2 Cone Mosaic Differences. We still need to develop tools to estimate the S-cone free zone and other properties of the foveal cone mosaic reliably. I think its just a matter of time, and I'm quite sure that however it is accomplished, adaptive optics will play a key role.

SC: We have done some work on imaging cone photoreceptors in eyes with different severity of myopia. We also show decrease in cone density (or increase in cone spacing) with an increase in severity of myopia. This is in agreement with previously published results. This finding is attributed to elongation of axial length associated with myopia development. This finding is further supported psychophysically by measuring the Stiles-Crawford Effect of the first kind (SCE-I). We found both the rho and the peak position of the SCE-I function in myopic eyes to be lower and shifted nasally respectively compared to those of emmetropic eyes, and the extent of these changes increased with an increase in myopia. This finding further supports the notion that retinal stretching caused by myopia is physically pulling the cones away from the center of the pupil. Separating S-cone mosaic from L and M cones in myopic eyes is an interesting idea. I wonder how this would be related with the effect of increased outdoor time in slowing down the progression of myopia.

AH: I don't have a direct comment/answer but given some evidence that modulating contrast (e.g. SightGlass), and using different wavelengths/spectral bands (although the results are confusing as red/blue seem to have exhibited positive and negative effects from different studies), seem to influence short-term indicators of myopia progression, perhaps investigations into the retina receptors should expand to include those other dimensions (e.g. wavelengths) and also include the post-retinal processing effects.

RE: The S-cone mosaic individual differences seem to me to be crucial. Unless the role of S cones (mosaic and sensitivity) can be ruled out -- and I don't believe it will be -- I think this should be one of the primary emphases in myopia research.

*5.- Axial biometry in the most distinct feature of myopia, and **choroidal thickness** has been pointed as a potential immediate biomarker to longer-term axial elongation.*

* What physiological factors contribute to choroidal thickness and how is it modulated with defocus or optical manipulations?

* Most data in the literature have been obtained with off-the-shelf low coherence interferometry instruments, and in real time as the stimulus was presented? Besides, the magnitude of the reported thickness changes is close to the axial resolution of the optical coherence tomography technique. What technical advances would improve choroidal thickness measurements and the study of its implications in myopia?

MW: With reliable volumetric reconstruction of the choroid, it is possible to assess the degree of decorrelation of rich choroidal paterns in the sattler layer, which vary in shape at different depths. The decorrelation between vascular patterns measured with retinal stimulation can be a sensitive (subresolution) tool for detecting changes in the geometry of the choroid itself. Such capabilities are offered by the spatio-temporal optical coherence tomography (STOC-T) technique.
Auksorius, E., Borycki, D., Wegrzyn, P., Sikorski, B.L., Lizewski, K., Zickiene, I., Rapolu, M., Adomavicius, K., Tomczewski, S., and Wojtkowski, M. Spatio-Temporal Optical Coherence Tomography provides full thickness imaging of the chorioretinal complex. iScience, 25, 12, 105513, (2022)

JR: Q1: Defocus can induce local changes in choroidal thickness by adjusting the volume of blood in the tissue. This process occurs relatively quickly.
Q2: Adaptive optics OCT may be able to do this.

AR: Q1 Factors: From what I've learned about this so far, it seems that a lot more research needs to be done to validate the use of choroidal thickness as an effective biomarker. It will be tremendous for the field if it is.

Q2 Advances: There is much room for technical advances on several fronts, 1) Making devices more affordable to reach a larger population through screening, especially for underserved populations. 2) Measuring axial length across the visual field quickly and accurately 3) Using smarter analysis tools to make measurements faster and more robust.

MC: Choroidal thickness is influenced by a range of physiological factors – some of the topics studied to date have been optical blur, accommodation, circadian rhythms, autonomic nervous system, cardiovascular system, exercise, caffeine, nicotine, antimuscarinics, image polarity, and light intensity and wavelength. How the choroid changes thickness in response to optics is thought to be due to changes in vascular (lumen) area which reflects changes in blood flow, or possibly changes in tone of non-vascular smooth muscle. But this is still relatively unexplored. The changes in thickness begin within minutes, so it is likely that the vascular or non-vascular smooth muscle is responsible for the changes. OCT has better resolution than low coherence interferometry and to provide reliable estimates of thickness changes it requires retinal tracking and image registration and averaging of multiple measurements. More detailed studies of choroidal changes with swept source OCT angiography is one of the most promising avenues at the moment. Better imaging of the choroid would be useful to understand vascular and non-vascular changes in the tissue, along with better methods for quantifying blood flow information and an understanding of oxygenation of the choroid during these changes.
(Response discussed with Prof Scott Read, who is an expert in the choroid and myopia area.)

AH: The difficulty with studies on choroidal thickness changes is they typically find effects through inferential statistics averaged from large samples. The accuracy of instrument is as yet not sufficient for individual ('single point') measurements. This will hampen our ability to conduct more in-depth studies where intersubject variations in predictor variables are to be considered for a more 'customised'/'individualised' approach to myopia studies.

FM: Q1. am not that familiar with the biochemical processes, but there is probably a biomechanical component where stretching of the choroid results in some thinning.

Q2. To be frank, I am not convinced regarding the findings of choroidal thinning or changes in eye length with accommodation. In our own published studies (Chang et al) with high-resolution custom-developed and whole-eye OCT instrument we found no changes in eye length with accommodation. One limitation of the studies finding a change is that they show aggregate data. Form aggregate data it is not possible to conclude if the change is consistent across all volunteers or of there is variability in the responses. A SS-OCT with deeper penetration that can more precisely identify choroidal boundaries would help provide more precise measurements.

QUESTIONNAIRE 2. Optical blur as a trigger of myopia

Prof. Pablo Artal	Laboratorio de Optica<br>University of Murcia<br>pablo@um.es	**PA**
Prof. Linda Lundstrom	Biomedical and X-Ray Physics Dept.<br>Royal Institute of Technology, KTH, Sweden<br>chlinda@biox.kth.se	**LL**
Prof. Thomas T Norton	School of Optometry<br>University of Alabama at Birmingham, USA<br>tnorton@uab.edu	**TN**
Dr. Hema Radhakrishan	Biology, Medicine and Heatlh<br>University of Manchester, UK<br>Hema.Radhakrishnan@manchester.ac.uk	**HR**
Prof. Frances Rucker	New England College of Optometry, Boston, USA<br>ruckerf@neco.edu	**FR**
Prof. Frank Schaeffel	Section of Neurobology (University of Tübingen) Insitute of Molecular and Clinical Ophthalmology Basel<br>frank.schaeffel@uni-tuebingen.de	**FS**
Dr. David Troilo	SUNY Optometry, New York, USA<br>troilod@sunyopt.edu	**DT**
Prof. Geunyoung Yoon	School of Optometry, University of Houston<br>gyoon2@Central.UH.EDU	**GY**
Dr. Len Zheleznyak	Clerio Vision and Center for Visual Science<br>University of Rochester, New York<br>lzheleznyak@cleriovision.com	**LZ**

1.-Computer modeling, psychophysical experiments in humans presented with artificially blurred images, and experiments with animal models in chromatically manipulated background suggest that longitudinal ***chromatic aberration is a cue for emmetropization****, and emmetropes and myopes may respond differently to these chromatic cues.*

* What study could be designed that uncontestedly demonstrate the role of LCA on emmetropization?

* What open questions remain in understanding the role of LCA in detecting the sign of the focus (sparsity of S-cone mosaic versus higher density of L/M mosaics, others)? Is the mechanism driven by blur of red or blue?

HR. The adaptation of the visual system in myopes and emmetropes to LCA will be interesting to study by altering the LCA and seeing how the eye responds.

PA: Although is true that there have been traditional ideas on the role of chromatic aberration in emmetropization, in my personal opinion this not as clear. Chromatic aberration in the human eye

is very large, but the visual impact is very limited in general, so this could be also the situation with emmetropization. Additional experiments were chromatic aberration can be removed (for example using the Two-photon vision approaches) can be useful to better understand this important topic.

LZ: Q1: Recent studies investigating the impact of chromatic cues on accommodation, eye growth in animal models and short-term changes in human choroid thickness have been limited to computational methods (e.g. preferentially blurring the one of the RGB channels in a digital display). While this approach has produced some convincing results, it suffers some limitations. LCA affects the optical quality both at the fovea and in the periphery, but current methods have not appropriately addressed the periphery. Computational chromatic cues implemented in digital displays, are in so-called "intensity space", rather than "wavefront space". For example, digitally blurring the blue channel of a digital display does not produce a defocused wavefront for blue wavelengths, thereby preventing any possible interaction of chromatic wavefronts with the wavefront aberrations of the eye, leading to a non-realistic peripheral optical quality. This becomes especially important in the periphery, where astigmatism is a dominant wavefront aberration, and blur orientation becomes wavelength dependent. To overcome this limitation, diffractive optics can be used to manipulate chromatic cues. By changing the dioptric power of a diffractive wavefront, one can correct, reverse, or magnify the eye's LCA. This can be achieved with adaptive optics (AO) vision simulators equipped with a diffractive element (e.g. spatial light modulator) or diffractive contact lenses. While AO vision simulators have a limited field of view (several degrees), diffractive contact lenses allow for unobstructed natural viewing conditions, allowing the peripheral retina to also experience LCA-modulated conditions. In addition, contact lenses can be used in a longitudinal study, whereas AO vision simulators allow for limited visual exposure to a test condition (<3 hours).
What study could be designed that undisputedly demonstrates the role of LCA on emmetropization? A Randomized Control Trial in children, measuring the refractive error and axial length over time (1 year at least) in several test condition groups: o Control: conventional correction o Test Condition 1: Zero LCA – a diffractive optic used to correct the eye's LCA, removing this cue. o Test Condition 2: Reverse LCA – a diffractive optic used to reverse the eye's LCA. o Test Condition 3: Double LCA – a diffractive optic used to double the eye's LCA. • To deliver the chromatic test conditions 1-3, a diffractive contact lens8 could be used.

Q 2: We need to build a better understanding of the field-dependent chromatic optical properties of the emmetropic, myopic and hyperopic eye. LCA (i.e. wavelength-dependent defocus) interacts with the peripheral monochromatic aberrations, such as astigmatism, to change the shape and orientation of the peripheral point spread function as a function of wavelength. What are potential mechanisms of detection of chromatic optical cues in the periphery? What are the spatial and temporal properties of the color-sensitive detection mechanisms in the periphery (L, M and S cones, rods, intrinsically-photosensitive retinal ganglion cells)? Are the radially aligned receptive fields of orientation-sensitive retinal ganglion cells9 sensitive to optical blur orientation at a particular wavelength. What about when taking fixational eye motion into account (which has been shown to differ in refractive error groups)? TCA causes a wavelength-dependent retinal magnification. There may be an interaction between TCA, motion detection, and fixational eye movements. For example, shorter (blue) wavelengths have lower retinal magnification, meaning the retinal image is slightly smaller, as compared to longer wavelengths. Thus as a broadband object moves laterally across the eye's field of view, it's "blue" image travels slower than the "red" image (which covers a larger area on the retina).

DT: Although studies using pseudo monochromatic environments show that the spectral composition of light affects the visual regulation of eye growth, they won't do demonstrate the role of LCA. The study by Swiatczak and Schaeffel in 2022 comes close to showing that LCA affects

emmetropization; specifically, they report that when longer (redder) wavelengths are in better focus eye growth is reduced. The one caveat in this study is that the changes in axial length reported are likely due to choroidal thickness changes. The relationship between choroidal thickness changes and axial growth changes is not completely understood. Studies that use short term choroid thickness changes as a surrogate for axial eye growth changes need confirmation because of the variability in choroidal thickness.

GY: A study that could directly address the LCA question would be to investigate the response of the visual system to a condition under which the natural LCA in the eye is corrected and/or reversed while maintaining the natural light spectrum. Creating this condition is feasible in both a laboratory setting and daily life for short-term and long term studies, respectively. Open questions: To my knowledge, we don't have a good understanding (or consensus) of LCA's role in accommodation. Is it necessary for the visual system to have LCA in order to detect the sign of defocus? Are there other factors affecting the detectability of the sign of defocus (e.g. astigmatism, higher order aberrations, eye movements) that may interact with LCA? What is the role of TCA, especially in the peripheral retina?

TN: Q1. What study could be designed that uncontestedly demonstrate the role of LCA on emmetropization? Determining the role of LCA – mechanistically, how it guides emmetropization, will be more difficult than just establishing that LCA is a critical cue, which has already been done by removing LCA cues. Studies in macaque monkeys, tree shrews, chicks, guinea pigs and mice have found that immersing animals in narrow band light that removes LCA cues have found that this disrupts emmetropization. The eyes become myopic or hyperopic depending on the wavelength used (long, or short) and the species. This implies that LCA must be present for the emmetropization mechanism to function properly. Moreover, when animal eyes deviate from emmetropia in narrow-band light, substantial hyperopia or myopia can develop. Without LCA cues, defocus is not a sufficient cue to guide eye growth to emmetropia or to maintain eyes at emmetropia. Although a lack of LCA in narrow-band light produces ametropia in all tested species, it is important to note that the direction of refractive change in response to narrow-band short or long wavelengths differs across species. In primates (macaque monkeys) and near-to-primate tree shrews, long wavelength light causes slowed eye growth, producing hyperopia. In several other species, (mice, guinea pigs), that are more distant from primates it is short-wavelength light that produces hyperopia. The reasons for the species differences have not been determined but could involve the transmission of short (ultraviolet) wavelengths by the optical media, the wavelengths at which the photopigments have peak sensitivity and, in birds, the number of photoreceptors. The fact remains that LCA cues are necessary to achieve and maintain emmetropia. A recent study may provide a way to separate the effects of defocus vs. LCA on emmetropization. In broadband light, defocus and LCA are correlated. A plus or minus lens imposes a defocus cue but also changes which wavelengths are in better focus on the retina. Is there a way to decouple defocus and LCA cues? Gawne et al. (2022) selectively blurred the short (blue) wavelengths but not the longer wavelengths on a visual display presented to tree shrews. Thus, the display of Maltese crosses provided ample sharp images at the long wavelengths, limiting defocus cues. Animals exposed to this change in LCA with minimal change in focus developed significant hyperopia, as predicted by the Gawne & Norton (2020) model.

Q2. What open questions remain in understanding the role of LCA in detecting the sign of the focus (sparsity of S-cone mosaic versus higher density of L/M mosaics, others)? Is the mechanism driven by blur of red or blue? Several factors suggest that the relatively sparse array of SWS cones is sufficient to guide emmetropization. One is that low to middle spatial frequencies, not high spatial frequencies, are used to guide emmetropization. The blue cone array is sufficient to detect blur in the range of 1 to 3 cycles per degree, which may be sufficient to guide emmetropization. It would not make sense to rely on high spatial frequencies for emmetropization because they could not

provide guidance when images are out of focus. Emmetropization may not be guided just by blur on the red or blue cones, but by an interaction of image blur on the LWS (or LWS+MWS) versus SWS cone arrays. The opponent dual-detector model developed by Gawne & Norton (2020) in tree shrews and adapted to humans (Gawne et al., 2021) may be useful in investigating this issue. A key question for further research is to examine how bipolar and amacrine cell types in monkey or tree shrew respond to the interaction of long vs. short wavelength blur. This could be done with single cell retinal recordings or possibly with imaging techniques that monitor the membrane potential of neurons.

FR: Q1. What open questions remain in understanding the role of LCA in detecting the sign of the focus (sparsity of S-cone mosaic versus higher density of L/M mosaics, others)? The inquiry into whether the sparsity of the S-cone mosaic, in contrast to the more densely populated L/M cone mosaic, impacts the detection of the sign-of-defocus raises significant considerations. Despite the reduced spatial resolution of the S-cone mosaic due to its sparsity, there are compelling factors suggesting that this may not impede its role in detecting blur:

1) Optimal Signal at Lower Spatial Frequencies: Evidence in accommodation indicates that a chromatic signal from LCA is most effective at lower spatial frequencies (Mathews & Kruger 1994, Owens 1980), where blur gradients are less steep. This aligns with the need for these mechanisms to respond to stimuli with varying degrees of blur.

2) Sensitivity to Blue Light Stimuli: Both accommodation and emmetropization exhibit sensitivity to blue light stimuli for both achromatic and chromatic signals. The achromatic mechanism is dependent on the focal length of the wavelength (less growth with exposure to monochromatic blue light), while the chromatic mechanism is dependent on the relative contrast available to the long (L-) and short (S-) wavelength cones (increased growth with a low L-/S-cone contrast ratio). Blue light activates S-cones and melanopsin in the ipRGCs, as well as other cone types depending on wavelength and species. It is known that ipRGCs are spatially insensitive (Allen et al 2019), and the spatial resolution of S-cones peaks at low frequencies (Cavonius and Estevez 1975), suggesting their capability to respond to situations with more pronounced blur.

3) Sensitivity to Red Light Stimuli: Red light, in contrast, primarily stimulates the more densely populated L- and possibly M-cone arrays. These cones, with higher resolution, are likely more sensitive and adept at responding to smaller degrees of either chromatic or achromatic blur in addition to larger degrees of blur. This involvement suggests an additional role in fine-tuning responses.

Is the mechanism driven by blur of red or blue? Emmetropization responses to achromatic stimuli illuminated by red and blue monochromatic light sources have been observed in many species (see Rucker 2019 for citations). Axial growth is driven in the predicted direction in cichlid fish (Kröger and Wagner, 1996), chicks (Foulds et al., 2013; Rucker and Wallman, 2008; Seidemann and Schaeffel, 2002; Torii et al., 2017), guinea pigs (Liu et al., 2011; Long et al., 2009; Qian et al., 2013; Tian et al., 2019; Wang et al., 2011) and rhesus monkey (Liu et al., 2014). Exceptions were seen in chicks exposed to far red (665 nm) and UV light (383 nm) (Rohrer et al., 1992), in tree shrew (Gawne et al., 2017a, 2017b, 2018) and in rhesus monkey (Hung et al., 2018; Smith et al., 2015). In chick, the predicted axial inhibition in response to blue light is prevented by exposing the eyes to blue light in the evening, with daytime exposure to white light (Nickla et al., 2018). Red and blue light drive the emmetropization response in more extreme directions than in white light indicating that both colors have a role to play. A chromatic signal from LCA, which operates in broadband light and compares contrast in red and blue, has been shown to modulate the emmetropization response in chick (Rucker and Wallman, 2012; Rucker et al 2020), tree shrew models (Gawne et al 2022), and in humans (Swiatczak et al 2022). However, the predicted axial growth responses to the chromatic signals are in the opposite direction to those from the achromatic signals (blue monochromatic light inhibits axial growth, while a low L/S-cone contrast ratio increases axial growth). Detection of the chromatic signal in chick is dependent on temporal frequency and

contrast (Rucker et al., 2020) and some degree of broadband light is necessary for this chromatic signal and response. While the use of repeated low-level achromatic red -light therapy is currently a hot topic, the etiology of this effect is not clear. The observed reduction in vitreous chamber depth and axial length arises partially from transient choroidal thickening, an effect that has been confirmed in multiple studies (Salazano et al 2023). Similar effects of red-light exposure on vitreous chamber depth have been seen in macaque (Smith et al 2015) and tree shrew (Gawne et al 2017), coupled with choroidal thickening in tree shrew. Also in chick, choroidal thickening has been observed in longer-wavelength light with higher contrast (Rucker et al 2018; Watts et al., 2020; Lin et al 2019) and with evening exposures (Nickla et al., 2018). It is not clear whether the choroidal thickening in red light reflects the potential for long-term growth inhibition, whether it is a transient choroidal modification, or whether it arises from inflammation or phototoxic damage. The multifaceted nature of emmetropization, which involves antagonistic achromatic and chromatic signals from Longitudinal Chromatic Aberration (LCA), and their dependence on temporal frequency, contrast, as well as circadian influences underscores the need for further research and demonstrates the inadequacy of a simplistic dichotomy between red and blue stimuli.

LL: The main issue for me is whether the recent findings that Frank Schaeffel reports on red in focus with blue channel blurred (simulating LCA) also apply to a developing child's eye and are not just a consequence of the myopic eye being adapted to blur or LCA for that matter (His subjects were already around 24 years old on average I think). Studies need to be done on blur adaptation in humans - especially in the low sf blue channel compared to the higher sf L/M system.

*2.- **Peripheral hyperopic defocus** has been attributed to be a primary trigger of myopia, and a primary target for optical intervention, but the peripheral cues for the detection of the sign of myopia are a matter of debate. Besides, intersubject variability in peripheral retinal shape and refractive error is large, and whether excessive peripheral hyperopic blur is a cause for myopia (even before this is corrected) or a consequence of axial elongation is unclear.*

* What mechanisms of action, specific to peripheral retina, would underlie the detection of the sign of defocus and what studies could be designed to elucidate those mechanisms?

* It appears that baseline peripheral refraction in isolation does not predict the onset or progression of myopia, and it is only the relative peripheral hyperopic defocus with respect to the fovea which exacerbates axial elongation. Peripheral hyperopic defocus is emphasized with spectacle and single vision contact lenses, prescribed once myopia has initiated. But what triggers myopia in the first place? If peripheral hyperopic defocus precedes axial elongation, what makes "to be myopic eyes" more myopic in the periphery? Is it retinal shape? Or the refractive elements (cornea and lens) peripheral optical quality?

* Peripheral defocus at near could be a crucial factor, resulting from thinner crystalline lens and distinct accommodative responses in (becoming?) myopes. However, results are inconclusive regarding peripheral aberrations and refraction as a function of accommodation. How could this be disentangled?

HR: Peripheral refraction does not seem to change much between distance and near. The errors in the periphery are so large that the changes caused due to the change in shape of the lens do not have a major effect on the peripheral refractive error. Therefore, it is difficult to see how near work and peripheral refraction interact to produce myopia. The increase in light levels with outdoor activity is likely to have an impact on peripheral vision in leading to changes in eye growth. It will be good to see studies in the future which explore the interaction between peripheral refraction, near work, outdoor activity and lighting levels.

PA: Again, in my opinion peripheral defocus seems to be more a consequence of eye growth than and cause. We have been performed different studies in the last decade both in adults and in children and all the results suggest this statement. I know that there are other opinions and that several commercial products are based in this assumption, but I do not think is based in any solid research. Concerning the last question, it is possible to measure peripheral defocus and aberrations for near targets using some of the new devices developed. What will be important is to carry out these studies in children and not in young adults.

LZ: Q1: The peripheral retina's optical quality is dominated by oblique wavefront aberrations, such as astigmatism and coma. It follows that astigmatic blur orientation alternates 90 degrees in the presence of positive vs negative defocus. Previous studies have shown that retinal ganglion cells in rabbit12, cat13, 14 and primate15 are centered on the area centralis or fovea, depending on the species. Taken together, we can hypothesize that anisotropic neural circuitry may be sensitive to anisotropic optical quality, similar to a photonic quad-cell detector16, and may provide a cue for eye growth.
Q2: Due to the relative tilt of the anterior chamber, the peripheral retina experiences significant astigmatism and coma. In the presence of these wavefront aberrations, peripheral blur orientation alternates from radial to circumferential with relative peripheral myopia or hyperopia, respectively. Let's take the example of a distance-gazing emmetropic eye. It has, on average, small amounts of relative peripheral myopia. Its peripheral retina experiences radially elongated blur, due to the wavefront aberrations of defocus, astigmatism and coma. However, for near objects prior to accommodation, and to a lesser extent even after accommodation but in the presence of lag, the periphery is hyperopic, and thus no longer experiences radial, but rather circumferential blur. Does the poor visual diet of an indoor, near-rich, environment expose the retina to the "wrong" (i.e. circumferential) blur orientation? Wavefront optics says yes. Also note that dimmer indoor illumination will increase pupil size, exacerbating wavefront aberrations. It is possible that the biochemical cascade required for emmetropization is inhibited or disrupted (i.e. an irregular production of dopamine, retinoic acid, etc.) by the presence of the "wrong" (i.e. circumferential) peripheral blur. In the case of the myopic eye, even while distance-corrected and distance-gazing, circumferentially elongated peripheral blur dominates, potentially contributing to the on-going progression and further axial elongation. Said another way, before myopia onset, an indoor, near-rich environment exposes the peripheral retina to circumferential blur. After myopia onset and the globe has developed a prolate shape, the peripheral retina is also exposed to circumferential blur, but now both for far and near objects. We are not yet able to mechanistically connect (a) the eye's optics described above with (b) the biochemical cascade of retinal-to-scleral signaling. However, it appears there is a concomitance of circumferential peripheral blur with myopia versus radial peripheral blur with emmetropia and hyperopia. Unanswered questions remain, such as the role of spatial frequency, wavelength, and lateral and longitudinal temporal modulations due to eye motion and accommodative microfluctuations, respectively. Ultimately, a better understanding of the mechanisms driving eye growth will yield the development of more efficacious therapies for slowing the rate of eye growth.

DT: Q1. Early experimental studies showing local visual regulation of eye growthwas a paradigm-shifting finding that changed the way we think of emmetropization and myopia development. Subsequent experimental studies showed that imposing visual signals on the peripheral retina can affect axial growth and refraction, providing proof of concept that optical treatments imposing myopic defocus in the periphery could effectively change axial growth and reduce myopia progression. These findings supported the development of effective optical treatments that provide additional positive power on the peripheral retina while minimizing it centrally allowing for clearer axial distance corrections. The idea that peripheral hyperopic defocus is a primary

trigger of myopia is, however, an oversimplification for such a complex multi-factorial system. The central retina, where photoreceptor density and visual processing is higher, is surely also involved in visually regulated eye growth. Evolution would favor that the entire retina is involved and that signals across the retina related to the visual environment and visual behavior are integrated temporally and spatially to produce growth signals that shape eye growth adapted for its environment. The sum of local growth changes will affect overall global growth, eye size, and axial refractive state. This view is supported by findings that more defocus imposed across the retina has larger effects. It leaves open, however, the question of how the visual processing for eye growth control is weighted across the retina. Is it center-weighted because of cell density or peripheral-weighted because of greater area? Are there asymmetries across the retina in the gains of defocus-detecting retinal processing? Understanding the cell and molecular basis of the growth signals emerging from visual processing across the retina will help answer this question among many others related to the nature of visually derived growth signals that regulate eye growth and refractive state.

Q2. What triggers myopia in the first place? If peripheral hyperopic defocus precedes axial elongation what makes "to be myopic eyes" more myopic in the periphery? These are important questions that need to be answered to understand the development, and ultimately the control, of myopia. The experimental finding that imposed myopic or hyperopic retinal defocus (divergent or convergent defocus respectively) bidirectionally change eye growth to compensate for the defocus (originally demonstrated by Schaeffel et al.) raises the questions: Why does myopia occur at all? Why isn't it self-limiting? It is unlikely that visually regulated eye growth evolved to achieve emmetropia per se. More likely, what we call emmetropization is a process of visually regulated eye growth evolved to adapt the eye to the environment and the organism's visual behavior. So, it shouldn't be surprising that myopia occurs and even that it is prevalent because of visual conditions and behaviors in modern human environments. Why myopia progresses and isn't self-limiting is a complicated question. The short answer is that in most cases of low to moderate myopia, the emmetropization mechanisms may be at work under a set of environmental and visual conditions that adapt the eye to some myopia. This myopia is, in fact, limited by the conditions and as the eye reaches maturity. In the cases of progressive and high myopia, these mechanisms appear to break down and eye growth becomes dysregulated possibly because of disease or genetic conditions. What makes understanding why myopia progresses or not so challenging is that disentangling the many factors involved in the control of eye growth is difficult. Many environmental and visual factors known to affect eye growth and refractive state combine and interact during development. Environmental factors include light intensity, spectral composition, visual space, object proximity, spatial frequency content, and contrast. These combine with visual factors such as optical aberrations, retinal defocus, accommodation, eye size, shape, and symmetry. These are all further combined with visual behaviors that affect the temporal and spatial integration of visual signals across the retina that produce the growth signals which ultimately alter sclera extracellular matrix formation. The cellular machinery of the growth signals and their responses are themselves affected by their genetics and control of gene expression. In 1961, before visually guided local growth of the eye had been demonstrated, van Alphen proposed that the relationship between the overall growth of the whole eye and axial elongation are normally coupled proportionally during emmetropization but are decoupled in progressive axial myopia. During the early postnatal active growth of the eye, equatorial (horizontal and vertical) growth reduces optical power that is offset by increasing axial growth to achieve and maintain emmetropia. Any mismatch in the rates of equatorial and axial growth results in ametropia. Myopia typically results when axial growth continues as equatorial growth slows and stops. Van Alphen speculated that weakness in the sclera posterior to the limbus in the presence of high IOP, was the principal cause of continued axial elongation and myopia. In cases of progressive myopia, we now hypothesize that as the equatorial growth of the eye slows and total optical power stabilizes, visual conditions across the retina are responsible for keeping the posterior segment growing, increasing axial elongation and myopia.

Without the ability to reduce optical power further the myopia increases until the combination of conditions across the retina change and the axial growth slows. Optical treatments that impose myopic defocus on the retina, or generally interfere with the retinal signal to grow, will slow eye growth and reduce progression. The maturity of the eye and potential for postnatal growth is an important factor. Myopia onset at younger ages, when there is significantly more active growth of the eye, is associated with more progression and higher amounts of myopia. Most experimental studies were performed on fast growing eyes of infant and juvenile subjects. A few experimental studies demonstrated some regulatory growth in mature eyes, but with generally smaller effects, suggesting that visually regulated control of growth is more effective in immature actively growing eyes. Notably, studies demonstrating optical myopia management have mostly been conducted in children. Sorting age of participants and rates of pre-treatment progression to predict treatment efficacy need to be considered.
Q3. Peripheral defocus at near could be a crucial factor, resulting from thinner crystalline lens and distinct accommodative responses in (becoming?) myopes. However, results are inconclusive regarding peripheral aberrations and refraction as a function of accommodation. How could this be disentangled? Because of the possible relationship of near vision and myopia development, it is important to understand the relationship of retinal defocus across the retina during near vision and accommodation. But to fully understand how that might be related to myopia develop we must also understand accommodation behavior during prolonged near vision, the weighting of the visual signals across the retina (discussed above), and the temporal integration of the retinal signals that affect eye growth.

GY: My laboratory has proposed a new hypothesis that peripheral optical (blur) anisotropy could provide a cue for detecting the sign of defocus. This is based on evidence that (1) optical quality in the peripheral retina is characterized by changes in the dominant directionality of optical blur (due to significantly increased astigmatism and higher order aberrations in the periphery compared to the fovea), and that (2) successful optical interventions don't seem to cause the expected myopic shifts in the periphery (rather, they reduce peripheral blur anisotropy). These findings suggest that looking at simple refractive error may not be sufficient in explaining the intended mechanism. Thus, it is necessary to have a more complete understanding of the peripheral ocular optics. Although the meridional effect in the peripheral visual field is known, the cause is unclear. Whether visual experience with optical anisotropy has any impact on neural anisotropy and how this neural bias may affect the ability to detect the sign of defocus are intriguing questions to explore further.

TN: Why does emmetropization depend on signaling in the near peripheral retina? Location is a key factor. The macular area, sitting at the posterior pole, cannot control its location relative to the cornea. In mammals, if the eyeball enlarges due to scleral growth in the periphery, the fovea has to "go along for the ride." The half-eye studies have shown that retinally-generated emmetropization signals pass directly back through the RPE and choroid to control the scleral extensibility (or growth) in a region. This can occur throughout the retina, but the receptive fields of retinal neurons in the far periphery are too large to have the acuity to detect blur and generate useful emmetropization signals. If the fovea cannot control its location and the far periphery cannot produce useful signals, this leaves the near peripheral retina. So, the same retinal mechanisms may be occurring throughout the retina, but the near periphery is the only region that can produce useful signals to control axial elongation. We don't know how the sign of defocus is determined, but LCA occurs throughout the eye and (as noted in answer to question 1) is well suited to accomplish this task. That said, emmetropization is evolutionarily old and important, so there likely are multiple, redundant mechanisms involved.

FS. Finally, the role of foveal input is unknown. Right now, the 6-12 deg parafoveal area has been shown to be most responsive to defocus, but certainly eye growth is not only controlled between 6

and 12 deg. The fovea can probably not control it because of the tiny area that it covers in the fundus. In a recently released publication (Swiatczak et al 2024) we concluded that the fovea is not needed to generate a growth inhibiting signal for the eye, and the 3-9 deg annular parafoveal area seems to contribute most to it

LL. In relation to the periphery, one needs to distinguish between signals that facilitate eye growth and myopia, versus an inhibitory error signal (which may in fact be quite separate and not just a bidirectional single signal with down regulation of the growth side). The animal studies do not support that the signals are only in the periphery even though these are important, since the best classical inhibitory signals (real myopic defocus) include both central and peripheral retina - in animal studies, generally the bigger the area the more effective it is. The periphery is super sensitive to movement, yet there are almost no studies looking at potential cues with stablished retinal images, let alone specific to selective peripheral stimulation. Very few studies have varied temporal characteristics of stimuli - studies are needed on elucidating the typical temporal range for inhibitory growth stimuli. Is the inhibitory blue stimulus matched to ipRGCs temporal properties? As for accommodation, there is a great need for sophisticated studies in mammals, primates and humans where the actual blur on the retina is precisely measured. So far all we have is ancient studies in a grey squirrel (McBrien) which was using lid suture and changed cornea power or studies in chick which has large amounts of choroidal accommodation available (unlike other species which depend on lens) and retinal factors released during inhibition also act on choroid thus mudding the waters. One of the problems is that biology generally works by having multiple fail-safe mechanisms, so I would be very surprised if accommodation does not play a role. Perhaps accommodation may not be necessary, but may be still be sufficient under closed loop conditions.

*3.- The ability of the retina to **detect the sign of defocus** appears to be key in most theories of myopia development. In some scenarios (i.e. presence of hyperopic defocus, the equivalent to lens-induced myopia) the retina would be capable to detect the sign of defocus and grow accordingly. In some other scenarios, the mechanism for blur sign detection would be impaired in myopes, work differently for imposed positive or negative defocus in different refractive groups, be dependent not only of the spatial but the spatio-temporal characteristics of the optical blur, which would be altered by differences fixational eye movement dynamics (apart from optics and retinal shape) in myopes.*

* There at least two distinctive forms of blur (non-signed form deprivation) and hyperopic blur (negative sign) that trigger myopia. What other specifics of the blurred stimulus should be investigated and taken into account in the studies? Which spatial frequency range? Spectral content? Visual angle? Temporal dynamics?

* Is understanding the mechanism for detection and encoding of the sign of defocus, and its potential impairment, key to understanding myopia development? If this is the case, where the efforts should be placed.

* Should the question be reformulated on the origin (and specifics -temporal, spatial, spectral-) of the blurred signal falling on the retina and how this influences axial growth?

HR: In most animal studies, an animal that is shown hyperopic blur tends to become myopic and an animal with myopic blur grows hyperopic. Effectively showing that they have normal emmetropisation. In myopic humans, the emmetropisation mechanism is clearly not working and even myopic blur in the centre produces a myopic refractive error. It is important to understand how a myopic eye processes this sign of defocus (both in central and peripheral vision) differently

to a non-myopic eye. This might give an insight in to the underlying mechanisms which lead to the emmetropisation mechanisms failing in myopia.

PA: This is an interesting series of questions. First, I would like to point out that retinal blur is not only defocus blur. The structure of the retinal images is more complex that what is predicted by simple defocus. The combinations with the particular aberrations of the eye play an important role. In addition, color effects (as mentioned in your question 1) are also relevant and coupled. In addition, blur in the periphery will be dominated by peripheral astigmatism and coupled with defocus can produce strange behaviors. In summary, I would recommend a proper modeling and measurements of retinal blur.

GY: Some optical interventions have demonstrated efficacy in slowing down myopia progression. Results from interventional clinical trials have provided some clues about potential mechanisms of myopia development and progression, including blur anisotropy, retinal image contrast reduction, and increased depth of focus. Therefore, it is pivotal to investigate the impact that these interventions have on retinal image quality across the retinal eccentricity. In addition, the effects of adaptation to blur orientation via long-term visual experience are unexplored. I believe that advancing our understanding of how the visual system detects the sign of defocus is of crucial importance in relation to myopia development. It seems that the system disregards myopic (plus) defocus in the context of myopia development/control. It may be more appropriate to rephrase our question as "How does the system detect hyperopic defocus?", although with caution given that there has been evidence showing the bi-directional response of the choroidal layer to myopic and hyperopic defocus although what this response indicates in terms of long term eye growth. Peripheral optical characteristics (e.g. astigmatism and higher order aberrations) along different retinal meridians vary. Therefore, understanding the relative contribution of horizontal, vertical and oblique visual fields to myopia development (if any) is an important research topic, in addition to the significance of the size of the visual field.

TN: Q1. A type of blur that could be further explored is selective blur of short vs. long wavelengths, which minimally alters spatial frequencies, visual angle and temporal dynamics. As noted in the answer to question 1, just blurring the short wavelength (blue) channel of a computer monitor produces a stimulus that produces hyperopia in tree shrews (Gawne et al., 2022)
Q2. Understanding the mechanism for detecting and encoding the sign of defocus will be important for understanding myopia development for two reasons. One is that it will enable us to learn if the mechanism is impaired in children to develop myopia or high hyperopia. Another is that it will help to determine the key features in the visual environment that are used by the emmetropization mechanism to guide refractive development. It appears that something is missing in the modern visual environment that is causing so many people to develop myopia. If you know what factor(s) are driving emmetropization, you can make sure to provide those factor(s).

FR: Q1. Achromatic and Chromatic Signals: While asymmetric chromatic signals from LCA induced blur provide an instantaneous measure of the sign-of-defocus, achromatic signals with symmetric blur, require a "trial-and-error" approach. Since both signals are likely operating in broadband light, the accuracy of the emmetropization response may depend upon the weighting of the antagonistic achromatic and chromatic signals from LCA. In scenarios where the achromatic signal suggests ocular growth, prompted by the longer focal length of the red components of light, the chromatic signal simultaneously signals a deceleration of growth, when the L/S-cone contrast ratio is elevated. The dynamic interplay and weighting of these signals likely govern the set-point of the emmetropization mechanism, potentially serving as a homeostatic mechanism to prevent ametropia. Since detection of these signals depends on cone contrast sensitivity, temporal

sensitivity, spatial sensitivity, and circadian influences, it is not surprising that there are differences between groups.

Temporal Sensitivity: Temporal conditions induced experimentally support the hypothesis of antagonistic mechanisms controlling emmetropization. For instance, a low-contrast broadband (blue rich), high-frequency, stimulus with chromatic cues—mimicking hyperopic focus—leads to an increase in axial growth in chick (Rucker et al., 2020). Conversely, a high contrast, low-frequency, achromatic stimulus (blue rich), leads to reduced axial growth (Rucker et al., 2018; 2020), as long as the blue component is bright enough (Yoon et al., 2021). The same antagonism is seen with a chromatically modulated stimulus, a high contrast red/green light, modulated at a low temporal frequency, induces axial growth, while the same stimulus modulated at a high temporal frequency reduces axial growth (Rucker et al., 2018). Conversely, a blue/yellow stimulus reduces vitreous growth when modulated at a low temporal frequency, while it increases vitreous growth when modulated at a high temporal frequency. While the sign-of-defocus is determined by each mechanism in its own way, the overall response is likely to depend on the interaction of both systems.

Neural Mechanisms: Achromatic stimuli produced antagonistic responses from the parasympathetic and sympathetic nervous systems. The most significant reduction in axial growth in chick occurred when the achromatic stimulus mimicked emmetropia, when the stimulus had high frequency temporal modulation and high contrast, and when L- and M-cone contrast was equal and S-cone contrast was absent (Rucker et al., 2020). This response was mediated by the parasympathetic nervous system (Rucker et al 2023). It should be noted that the lens also thickens under these conditions. However, when the stimulus contains high contrast L-, M-, and S-cone stimulating components, lens thickening is mediated by the sympathetic nervous system, and lens thinning by the parasympathetic nervous system. More research is needed to determine the implications of this antagonistic neural activity and the etiology of the responses. Chromatic stimuli produced antagonistic responses with independent lesions of the two branches of the parasympathetic nervous system. Blue/yellow modulation caused a reduction of axial growth with lesion of the pterygopalatine ganglion, while red/green modulation caused an increase in axial growth with lesion of the ciliary ganglion (Rucker et al., 2022). If emmetropia is dependent on equal stimulation of these neural pathways by environmental stimuli, then it is likely that cone sensitivity is another important factor in emmetropization.

Myopic Sensitivity: Studies have reported the potential for reduced S-cone sensitivity in myopic individuals, particularly when assessed with S-cone isolation (Taylor et al 2018) but not with blue light (Swiatczak & Schaeffel, 2022). This reduction in S-cone sensitivity may impact the ability of myopes to detect both chromatic and achromatic signals from LCA. Diminished sensitivity in these signals could potentially compromise the accuracy of the emmetropization response, potentially leading to increased axial growth and myopia. Since chromatic and achromatic responses are governed by distinct neural mechanisms, factors influencing these mechanisms could bias the setpoint and degree of ametropia. In some other scenarios, the mechanism for blur sign detection would be dependent not only on the spatial but the spatio-temporal characteristics of optical blur, which would be altered by differences in fixational eye movement dynamics (apart from optics and retinal shape) in myopes.

Temporal dynamics: Temporal stimulation, whether induced by fixational eye movements, head movements, body movements, or external visual stimuli, may play a pivotal role in influencing the emmetropization process. A chromatic signal from LCA has been shown to modulate the emmetropization response in chick (Rucker and Wallman, 2012; Rucker et al 2020), tree shrew models (Gawne et al 2022), and in humans (Swiatczak et al 2022). In chick, environments characterized by low-contrast, high-temporal-frequency stimuli in broadband light (blue-rich) consistently generated chromatic blur signals from LCA, resulting in increased growth with a low L/S-cone contrast ratio and decreased growth with a high ratio. Conversely, visual environments featuring low-temporal-frequency stimulation, produced an accurate refractive response to

achromatic blur simulation, with reduced growth when blue contrast was high, and increased growth when red contrast was high (Watts et al., 2020; Rucker et al., 2020; Lin et al., 2019). Increased growth of the eye beyond the focal length of the optics, results in myopia. These findings underscore the intricate interplay between temporal stimulation, chromatic and achromatic signals, and the resulting impact on the emmetropization process, offering valuable insights into the mechanisms governing refractive development. In designing studies investigating the impact of blurred stimuli on emmetropization, several specific aspects merit consideration in addition to temporal frequency.

Spatial Frequency Range: Optimal emmetropization necessitates a broad range of spatial frequencies. The selection of spatial frequencies should align with the requirements of each of the achromatic and chromatic emmetropization mechanisms and the temporal frequency of the stimulus. Lower spatial frequencies may be more relevant when considering the impact of blur on the emmetropization response. Lower spatial frequency ranges may be particularly effective under conditions of reduced contrast, as the emmetropization mechanism is designed to operate optimally in blurred conditions (Schmid & Wildsoet, 1997). Conversely, higher spatial frequencies, which are more sensitive to blur, might be necessary to determine when the eye is closer to being in focus since higher contrast and resolution are required. Therefore, studying the impact of higher spatial frequencies, which have a steeper blur gradient, becomes essential.

Contrast Levels: Given that the presence of an LCA signal was observed at 30% temporal contrast but not at 80% contrast (Rucker et al., 2020), varying levels of spatial contrast should be systematically investigated for achromatic and chromatic stimuli to understand the impact on the emmetropization process. Recognizing that a blurred image will inherently reduce contrast, exploring the specific contrast levels that optimize each of the emmetropization cues is crucial, but temporal effects need to be taken into consideration, as the growth of the eye is differentially sensitive to contrast at low and high temporal frequencies (Rucker et al., 2020; Watts et al., 2020).

Circadian Influence: Time of day of light exposure affects the ocular growth responses to spectral stimulation. For example, steady blue light inhibits eye growth in chick during the daytime but increases eye growth with evening exposures (Nickla et al., 2022). The influence of temporal frequency, spatial frequency, and contrast under these conditions is unknown. Considering these factors comprehensively will contribute to a nuanced understanding of the interactions between circadian rhythms, spatial frequency, contrast, and temporal frequency in the context of emmetropization responses to blurred stimuli.

Sensitivity of the human eye to the artifacts created by LCA: The chromatic signal from LCA creates artifacts at luminance borders. Sensitivity to these artifacts may differ among subgroups, and lead to difficulties in using the chromatic signals for defocus that arise from LCA. Measuring sensitivity to the artifacts created by LCA, as a means of measuring the sensitivity of individuals to chromatic signals from LCA, would provide insights into why some people develop myopia while others do not (Taveras-Cruz & Eskew, submitted 2023).

Q2-Q3. As research has progressed, it has become evident that the regulation of refractive development is a multifaceted process involving various controlling mechanisms. Emmetropization can function in monochromatic light, with achromatic stimuli from LCA, but it functions more accurately in white light, with the addition of chromatic signals from LCA. In addition, the setpoint is affected by temporal frequency of the retinal stimulation, and by disruption of circadian rhythms, as well as light intensity. The mechanism is clearly not the single mechanism control process described in earlier accounts. Efforts to mitigate myopia progression have faced challenges in achieving significant changes in growth rates, highlighting the need for a more comprehensive and holistic approach. Recognizing the diverse factors at play—ranging from spatial and temporal considerations to circadian and spectral influences—is crucial for a more nuanced understanding of refractive development. Such an approach acknowledges the dynamic nature of vision and refractive processes, paving the way for more effective interventions and strategies to address myopia and related issues.

FS: We have still no clear idea how the retinal image is analyzed to extract the sign of defocus over time, as needed for emmetropization. We don't know which retinal neurons are involved (perhaps not the ganglion cells) and what they exactly do. We do have patchy findings about the roles of ON/OFF pathways, L cones, chromatic aberration and luminance but could not yet design a model that predicts choroidal responses and eye growth in detail. I believe that the myopia problem could be solved without any drugs by simply stimulating the retina in the right way that it starts thickening the choroid – like changing the appearance of the computer screen, for instance adding myopic chromatic aberration.
A big question is what has changed in the myopic retina that it can no longer generate a growth-inhibiting signal – obviously, myopia does not inhibit itself as one would expect from a closed loop feedback system. We found that a number of responses are different in the myopic retina, like little response to positive defocus, to longitudinal chromatic aberration and to long wavelength light (which normally inhibits eye growth). Second big question: when does it change – before myopia starts or during myopia development.
Furthermore, a big unknown remains the role of accommodation. Consider that defocus imposed by a lens causes rapid changes in eye growth but defocus imposed by accommodation errors has almost no effect. How does the retina know the difference?

LL: I think the question should be reformulated to study not what causes myopia or the sign of defocus, but instead what visual error signals inhibit eye growth, which may be quite separate to those that promote eye growth. To complicate matters, some of these signals may be non visual (as they occur in darkness) and/or relate to the state of the other eye. (See eg Zhu X, McFadden SA. Chick Eyes Can Recover from Lens Compensation without Visual Cues. Optom Vis Sci. 2020 Aug;97(8):606-615.) So it is important to use closed loop conditions (eg Positive lenses) rather than recovery from deprivation myopia.

*4.-**Animal models of myopia** are an extremely valuable source in myopia research, because they offer the possibility of optical, pharmacological and genetic manipulations, in a relatively fast timeframe. Despite some differences in response across species, the induction of myopia through form deprivation and through negative lenses, and eye shortening with positive lenses is fairly universal. Also, the response tends to be local in the retina. But, do they relate to the observations in humans?*

* Why simple undercorrection (positive lenses) has not functioned (in the majority of reports) as an inhibitory treatment for myopia in humans? Should this be revisited with new studies?

* In animal models, the retina responds locally (modulating axial growth) in the areas exposed to blur. Why modulating peripheral focus in humans would control axial growth in the fovea?

HR: Animal models are great in showing how emmetropisation works but this has clearly failed in human myopes. We need to understand what has led to the emmetropisation failing in myopes and to this end. I always find the data from the outliers in the animal studies (where the animal eye has behaved differently to the majority) to be providing interesting data and may be these animals might be able to help us learn what makes emmertopisation fail.

DT: Q1. The use of experimental animal models to study emmetropization and myopia is based on the comparative method. Understanding the similarities and differences between species in the development and adaptative significance of a system provides a better understanding of the evolution of that system. The visual regulation of eye growth is no different, and discoveries in experimental models have led to deeper understand and more effective treatments in humans.

The treatments can be refined with well-designed controlled clinical studies and modified with new insights from additional experimental work.
Q2. Why simple undercorrection (positive lenses) has not functioned (in the majority of reports) as an inhibitory treatment for myopia in humans? Should this be revisited with new studies? I suspect that this has to do with visual behavior. If under corrected subjects view near targets closer, the amount of myopic defocus on the retina will be diminished. In animal models, the retina responds locally (modulating axial growth) in the areas exposed to blur. Why modulating peripheral focus in humans would control axial growth in the fovea? . How local peripheral effects can affect axial growth is discussed in detail above.

GY: Identifying a short-term biomarker is an essential step in advancing myopia research effectively and objectively. Biomarkers must provide clear insights into the relationship between the short-term response to an intervention and its long-term implication for axial elongation. One challenge is that study conclusions from animal studies seem to depend on the types of animal models used. This further complicates our ability to translate what we have learned from animal studies to human studies.

TN: Q1-2. In an important way, having an animal wear a positive lens differs from fitting a child with an undercorrecting lens – the animal wears the lens all the time whereas children can remove their lenses. Schmidt & Wildsoet found that intermittent plus lens wear somewhat reduced the induced hyperopia, though the effect was not as powerful as if a negative lens were removed for similar time period. However, this effect probably is not strong enough to explain the typical lack of success of undercorrecting children.
Q3. In animal models, the retina responds locally (modulating axial growth) in the areas exposed to blur. Why modulating peripheral focus in humans would control axial growth in the fovea? If the periphery has myopic blur and this locally affects sclera, all around the periphery but sparing the fovea, the fovea cannot move away from the cornea all on its own. Its location is determined by the periphery as noted in the response to question .

FR: Q1. The congruence in results observed across various animal species concerning lens-induced defocus, form-deprivation, physiological responses to light intensity, and pharmacological reactions to substances like atropine suggests a high degree of similarity in the emmetropization mechanisms. Given the well-preserved retinal cellular organization and neural circuitry across species, along with a consistent consensus among different organisms, the essential function of emmetropization mechanisms appears to have been broadly conserved throughout evolution. Consequently, there is a compelling likelihood that these findings can be extrapolated to humans.

Q2. More recent observations highlight the need for a comprehensive re-examination of the effects of positive lenses on humans, considering the broad spectrum of signals that influence the emmetropization response, including temporal, spatial, spectral, and circadian factors. Consideration of environmental factors: When positive lenses are worn indoors, exposure to clear retinal images from proximal objects may limit the blur experienced, especially with small amounts of under-correction. The indoor environment, characterized by limited spatial and temporal stimulation, reduced light intensity and restricted spectral output of the light sources, might impact the efficiency of the under-correction therapy. Signals for defocus from LCA, as demonstrated by Rucker et al 2020, function optimally with a broad range of contrast in the retinal images, and with a broad range of temporal stimulation, likely more prevalent in outdoor environments. Indoor illuminants are typically low energy in the short-wavelength range, making the lighting warmer and more welcoming, and the intensity is low compared to outdoor lighting. Yoon et al 2021 have shown that higher intensity blue light is necessary for the achromatic blue light component to exert its growth inhibition effects in chick. In addition, the influence of circadian effects, which differ

from daytime responses, further adds complexity. Perhaps, the optimal lighting conditions need to be reviewed for under-correction treatments. In addition, consideration of circadian influences becomes crucial in accurately interpreting the impact of treatments on emmetropization.

Q3 Why modulating peripheral focus in humans would control axial growth in the fovea? One hypothesis that could be proposed, is that the response of the more achromatic periphery of the retina is weighted by the response of the more chromatic fovea, introducing an intriguing dynamic in understanding the emmetropization process. This hypothesis, posits that creating clear images in the periphery could result in a robust achromatic signal for growth retardation, potentially surpassing the influence of chromatic signals originating from the fovea. However, the loss of the chromatic signal would inhibit fine tuning of the emmetropization response, potentially leading to perpetual increases in ametropia as growth inhibition leads to increased weighting of the chromatic signal for hyperopia. This conceptual framework highlights the interplay between the achromatic and chromatic components across different regions of the retina, emphasizing the potential dominance of the achromatic periphery in influencing the achromatic emmetropization response.

LL In relation to animal models, studies clearly show that blur in the periphery affects central retina. The local studies are generally hemifield, so not really "local". Nor has anyone looked carefully at the effects of hemifield deprivation on central change, but it is likely to still occur.

QUESTIONNAIRE 3. Environment and Myopia

Prof. Rigmor C. Baraas	Optometry and Vision Science<br>University of Southeastern Norway<br>rigmor.baraas@usn.no	**RB**
Dr. Ranjay Chakarboty	School of Nursing and Health<br>Finders University, Australia<br>ranjay.chakraborty@flinders.edu.au	**RC**
Prof. Ian Morgan	Biomedical Science and Biochemistry<br>Australian National University<br>ian.morgan@anu.edu.au	**IM**
Prof. Lisa Ostrin	School of Optometry<br>University of Houston, USA<br>lostrin@Central.UH.EDU	**LO**
Prof. Mark Rea	Icahn School of Medicine Mount Sinai<br>Mark.Rea@mountsinai.org	**MR**
Prof Richard A Stone	Perelman School of Medicine, University of Pennsylvania<br>stone@pennmedicine.upenn.edu	**RS**
Dr. Fuensanta Vera-Diaz	New England College of Optometry Boston,<br>vera_diazf@neco.edu	**FVD**
Prof. James Wolffsohn	School of Optometry<br>Aston University, UK<br>j.s.w.wolffsohn@aston.ac.uk	**JW**

1.- *Understanding which* ***attributes of the outdoor environment*** *are most relevant in preventing myopia development is crucial to unravel the causes for myopia and design interventional strategies. Those factors would include light levels, light spectrum, spatial frequency content, contrast, distance of gaze, pupil diameter or type of activity.*

o What studies can be designed to test the effects of individual factors on myopia control?

o Should those studies involve field tests in real environments, or could laboratory testing effectively shed insight to those questions?

JW: Refractive error changes too slowly to reliably change a child's outdoor environment to examine the effect without too many uncontrolled confounding factors. Hence the best design we have is to examine choroidal changes as a surrogate measure. Animal studies have not proved reliable in mapping onto human findings. Real-would data is important, but even with large data, confounding data is difficult to fully account for.

IM: In general, it is hard to design human studies that isolate specific factors, and hence animal studies are more likely to give precise answers. However, in the case of time outdoors, it was

actually human studies that first made this a real possibility. Rose et al (2008) put forward the hypothesis that brighter light outdoors might be the important factor, based on previous data that showed that light was brighter outdoors, that brighter light caused more dopamine release from the retina, and that dopamine agonists slowed axial elongation. This hypothesis was tested in animal experiments by Ashby and colleagues, confirmed that increased light intensity largely suppressed the development of experimental myopia. Several other studies have supported this idea, but one influential study on non-human primates suggested that this might not be true for the LIM rather than the FDM model. Overall, however, the evidence favours the idea that brighter light slows the onset of myopia. The evidence suggests that this should also work in human eyes, and I think the evidence therefore suggests that this is likely to play a role. There are other hypotheses. One is that Vitamin D is important, and this one is hard to distinguish from bright light, because in general outdoor exposures during daylight hours bring both brighter light and more UV. However, Mendelian Randomisation experiments and some other detailed analysis has tended to suggest that Vitamin D is not the key. Only a RCT with Vitamin D administration would answer this definitively. Several other hypotheses have been floated. One was that city environments with bright light at night might be important. But this tends to fail because there are many cities around the world that do not have epidemics of myopia, and the correlation is much better with education. Recently Flitcroft has argued that the lower high spatial frequency content of indoor environments might be important, but the results of a test of this hypothesis in schools, while not yet published, is completely negative. Flitcroft has also suggested that the greater dioptric uniformity of outdoor scenes might be important, but as far as I am aware, there is no known mechanism for detecting dioptric uniformity. He has also suggested that the pattern of hyperopic and myopic defocus outdoors is likely to be different to that indoors, and that this may be important. This is soundly based on effects of hyperopic and myopic defocus in animal experiments, but it is hard to test, and does not lead to any particular intervention apart from increased time outdoors. Hagen et al have argued that because the prevalence of myopia is still low in Norway, daylight exposures cannot be important. However, even in mid-winter, there are still 6 hours of daylight, and this is enough to control myopia. Some of the logic in this paper is flawed, because it argues that children in Norway and Singapore appear to have similar exposures to light at the age of about 12, yet the prevalences of myopia are very different, and concludes that daylight exposures cannot make the difference. However, it ignores the fact that it is known that daylight exposures in preschool and early primary school children in Singapore are particularly low, and it is during these stages that myopia appears. The original Sydney-Singapore comparison showed that Chinese Australian children got much greater light exposures and had much less myopia than those in Singapore. Children of European ancestry were exposed to even more light, and had even less myopia. The paper by Hagen et al showed that children in Norway in the preschool and primary school years were exposed to a lot of light, and I would argue that this key difference invalidates their conclusions. Returning to the beginning, I would argue that we are most likely to get precise understandings of exposures from animal experiments. Factors such as spectral composition and circadian rhythm seem to have effects on development of myopia. So we then have to ask the question of under what conditions would these factors vary in human environments, and are these conditions associated with myopia. On the whole, only light intensity fits the evidence well. This does not preclude other factors playing a role, or being useful in myopia prevention. But Flitcroft has tended to argue that "time outdoors works, but it is not light." I can't see any scientific basis for this conclusion.

RS: Any of these proposed factors could be productively explored since each has been proposed and the role of each is imperfectly understood. Important additions could be season, time of day, latitude, properties of the ambient lighting and possibly the qualities of the phases of twilight. Real environments are most likely to provide meaningful results although laboratory studies in children and young adults, as well as in animals, could help formulate clinical hypotheses needed to effectively design clinical research.

FVD: I am providing this answer for studies on myopia control treatments, but also for studies on the etiology of myopia since we need to develop better methodologies for those as well. Besides those factors listed above, it is important to consider temporal aspects of vision. These are different in outdoor environments than indoors, not only because of the different nature of the activities we perform, but due to the environment itself. Eye movements are different in outdoor environments, which causes differences in retinal images, and there might be differences between children who develop myopia and those who do not, but this is yet to be determined. In addition to eye movements, being different, the dynamics of the visual environment are different in outdoors environments. This influences not only the images received but also the amount of light. When we are outdoors, the amount of light we receive changes frequently and by large amounts when a cloud passes by or we are under a tree shade (130,000 lux to 10,000 lux). This patten of light exposure is also important, in addition to the intensity and spectrum of the light source. In terms of spatial characteristics of outdoors environments, we need to ensure we consider the 3D of the visual environment, and across the entire visual field. Lastly, in terms of individual characteristics, pupil size is important, but we should also consider the individual's peripheral optics and accommodation. Note that studying pupil sizes is difficult since there are many factors, including emotions, that affect the sizes at each particular moment in time. Given all these considerations, the study of the effects of outdoors in myopia will likely need to be performed in real outdoor environments, since it is not feasible to duplicate all these factors (even the spectral composition of the sun, we are not yet close to duplicate that). Therefore, studies on children (highlighting children) before they develop myopia and children using all modalities of myopia management treatments while they are exposed to different outdoors and indoors environments are needed. We currently have devices that can measure light intensity, and to a certain extent, light composition, and viewing distances (again to a certain extent), but we do not have devices to measure all the factors listed above simultaneously. Developing such devices should in my opinion be a priority from our industry partners.

LO. It's a great question. Some of these optical and non-optical factors in the outdoor environment and their effects on myopia onset/progression are well-documented. One great example is time spent outdoors, which is now used as a clinical intervention for myopia prevention and control in children (see excellent reviews) [1-4]. Clinical trials show that an additional 40–80 minutes of outdoor activity per day [5, 6] and elevated light levels in schools using modified lighting systems [7] significantly reduce myopia onset and progression in school children. Recently, short-term exposure to moderate (500 lux) to high-intensity light (1000 lux) was shown to significantly slow axial elongation in humans [8]. Some recent studies demonstrate significant effects of short-term exposure to narrowband monochromatic light on axial length and choroidal thickness in human subjects [9-11]. However, this research is still in its early stages and necessitates further investigation before clinical translation. Similarly, another important factor might be the time of day of light exposure [12], which could potentially be used as an intervention for myopia control. Other factors, including reduced peripheral retinal defocus, increased depth of focus due to pupil miosis, relaxed accommodation, and an increased amount of high spatial frequency outdoors, have all been hypothesized to play a role in mediating the protective effects of the outdoor environment on myopia. However, these factors have not been thoroughly investigated. Much research is needed to understand not only their individual roles in myopia protection but also how they interact with each other in both outdoor and indoor lighting environments, to explore their potential use for myopia control interventions.

RB. Designing studies that would allow for more accurate and dynamic measures of the environment the children live in and how they behave in this environment – and making comparisons across different countries of the world would be very useful. This will require further

innovation in miniaturization of sensor that are capable of more accurate and dynamic measures than what is available today. This will also require that we develop an understanding of and agree on how we might categorize the child's physical environment, from being natural to being designed and configured for a particular type of behaviour – or if it is enough to classify in terms of spatial frequency content. The variation of spectral composition throughout the day and the year appears to play a role even in normal physiological eye growth. Thus, in a simple sense, a main thing to understand in relation to what may be protective of myopia as opposed to myopiagenic is frequency and dosage of daylight with variable spectral composition. Laboratory experiments with monochromatic light may seem a parsimonious way to approach it, however, this is not what the eye was evolved for. In addition, how an eye responds to the spectral composition in terms of eye growth may also relate to the spectral sensitivities of the specific eye's retina. Thus, there is a need to gain more knowledge about genes that may affect cone photoreceptor sensitivity, in relation to how this affects signalling of both colour and contrast – and consequently the signalling cascade that propagates to the sclera. Increasing our knowledge about environmental factors are essential, but myopia is a complex heterogeneous disorder with a multi-factorial aetiology. There is reason to assume that there are several genes that are inherited independently – altering genetic pre-disposition. As such, we need to make advances in understanding gene x environment interactions

2.- *Conflicting results on the relationship between near work, indoor vs outdoor timing and myopia are likely connected to the lack of precise* ***objective measures*** *of far/near viewing behavior, and of objective characterization of the physical properties of the visual environment (i.e. light exposure, spectral properties, viewed stimulus content and viewing distance, and duration and intermittency of the exposure).*

o What (wearable?) recording devices are most suited for the continuous recording of real time interactions of test individuals with the visual environment.

o Which strategies (AI?) can be used to navigate potential massive data sets that integrate all that information?

JW: None of the current wearables truly assess visual input rather than general environment. Even spectacle mounted cameras or range finders don't account for peripheral flow and eye movements.

I**M:** I think devices like Clouclip have the greatest potential, because they measure along the optical axis. The relationship between what a child sees, and what an instrument reads, is problematic with any other device.

RS: I suspect that those who have used recording devices have tried hard with the available measuring technology, but the reports I have seen have limitations because of directional light sensitivity, effects of clothing, recording light from a non-ocular location, etc. Novel devices that can measure viewing behavior, coupled with the viewing environment, might be useful; but they would be challenging to design and implement. Complex bioinformatics analyses will be necessary to deal with any large data set. Challenges for classical AI applications arise because of the long course of refractive development and uncertain endpoints (e.g., refraction, axial length, ocular shape, etc.) and the potential interactions of ocular parameters that may vary between subjects. Another complex area, very inadequately studied, is the mechanism(s) of the linkage of refractive errors to serious diseases in adulthood, such as glaucoma, various retinal disorders and certain types of cataract.

MR: The Daysimeter that we developed (Andrew Bierman *et al*. The Daysimeter: a device for measuring optical radiation as a stimulus for the human circadian system *Meas. Sci. Technol.* 16 2292 (2005) measures spectral power distribution and activity. From those data we can parse out the following: Light level, light spectrum, and activity. From those we can measure, on an individual basis, light exposure (duration and amount), spectrally weighted light exposure, time of light exposure, diurnal contrast (day vs night) and circadian entrainment (e.g., phasor analysis; Rea et al 2008). We can use a regression analysis to see how these factors vary between cohorts (e.g., urban vs rural). We have developed models of human (Rea et al 2021) and rodent (Bullough et al 2005) circadian-effective spectral and absolute sensitives, so both laboratory and field studies can be conducted to determine which factors affect each species.

FVD: I have mostly answered this question above. There are a few devices available in the market to measure light intensity, which is then used to estimate whether the subject is outdoors or indoors. This technique has limitations, as we assume that a luminance over 1,000 lux means the person is outdoors, which is not always the case. Some devices are in used as pendants, others as watches or clip to the spectacle frame. It is important that the device is located near the child's eyes, to more closely obtain objective data on what the child's eyes is actually exposed to. We will be presenting data at ARVO on comparison of two of the devices used in spectacles (Clouclip and Vivior). We certainly need devices that can five us comprehensive metrics of the visual environment, not only light exposure and/or viewing distance. I am not an expert in AI, but it seems to me that before we can use all the emerging, and very useful, AI techniques, we need more data. We simply do not have (enough) data.

RB. Q1. I would argue that these devices do not exist yet. For example, the range and accuracy for measuring luminance and spectral properties of current devices is not good enough. Another major obstacle is related to the size of these devices and where and what they are mounted on/to for continuous wear. Devices that need to be fit to eyeglasses are not suitable for children who do not need to wear eyeglasses. There are reasons to believe that making children wear eyeglasses they do not need may affect their behaviour. It is also challenging to monitor and/or convince teachers and parents to help monitor this. There is documentation showing that sensor worn on the wrist or around the neck give different measures than closer to the eye (as expected). There are also challenges with such devices when a child needs to put on a thick winter coat and gloves/mittens in winter. As I mentioned above, there is a need for innovation in miniaturization of sensor that are capable of more accurate and dynamic measures than what is available today — and that can be fitted and worn closer to the eye without the need to wear eyeglasses. This is the reason why we are organising an OPTICA incubator on the topic in February:
https://www.optica.org/events/incubator_meetings/2024/optica_incubator_on_wearable_dosimeters_and_light/ .

Q2. In terms of strategies to navigate large data sets, it is important to ask the right question based on the available data and its level of accuracy. Mendelian randomisation and regression discontinuity analysis are methods that are becoming and will continue to be useful, but this will require more accurate data for making reliable predictions. There are large between-individual variations, and strategies to navigate large data sets, also AI, should aim to contribute to the development of personalised myopia control. Thus, moving forward it is important to improve accuracy of collected data – and this relates to both how we collect behavioural and environmental data, as well as measures of refractive error (sufficient depth of cycloplegia) and including measures of ocular biometry (and to understand the properties of the crystalline lens this will also require sufficient depth of cycloplegia.

3.-· The relatively low prevalence of myopia reported in epidemiological studies in areas as diverse in latitude as Scandinavia, Australia or Tropical Brazil suggests that **outdoor light levels** may not be the predominant factor in myopia prevention, but probably other associated factors (lifestyle, lighting in classrooms, illumination during near work) are more important.

o Do you think comparative studies across different regions of the world using the same methodologies will shed light into the impact of sunlight on myopia?

JW: Outdoor light levels are only one factor, but so many factors change across regions that it would be difficult to account for all factors.

IM: have already dealt with this issue in discussing the paper by Hagen et al. However, there is more to add. This paper also did not discuss myopia in Eskimo/Inuit. Here, prior to the onset of schooling, there was little myopia, although the children were living in environments where there was as little as 1 hour of daylight in mid-winter. However, when the populations were moved into settlements, and children were given pretty rudimentary education, the prevalence of myopia rapidly increased. This suggests that the regulatory system is robust enough to cope with severe light deprivation for a few months in every year, when there are minimal educational/near work pressures, but that they system fails when these pressures are increased, even if they fall well short of the levels of pressure typical of East Asia. Going back to the two papers that really triggered this off, Rose et al showed that time outdoors was able to over-ride the effects of high levels of near work. Jones et al showed that time outdoors was able to over-ride the impact of parental myopia, which is certainly not a purely genetic effect. Thus right from the beginning, the idea was one of balance, rather than an exclusive emphasis on time outdoors. However, when talking about prevention, how easily a causal facto can be modified is also important. Giving children less education is really not an option, although shifts away from rote-learning may make educational more effective, reducing the hours required. But, getting children outdoors is much more feasible, and indeed China is currently trying to reduce educational pressures in the early school years, while getting children outdoors. It will be a few years before we see the real impact, but Taiwan is already having considerable success by increasing time outdoors alone. What does lifestyle mean? Can we increase light in classrooms? Yes, can we do it enough to get useful effects. The evidence is mixed. Comparative studies are difficult because if there are two arms, educational pressures and time outdoors, it is hard to compare like with like.

IM: The problem here is that the dopaminergic and melatonin systems are tightly linked, with increased dopaminergic activity likely to affect circadian rhythms, and disruptions to circadian rhythms likely to affect dopaminergic activity. If there is an association between sleep and myopia, it is because lack of sleep causes myopia, or because children who study late at night have disturbed sleep, but are myopic because they study late at night. Causality is key here. Modifiability is also important. How well can we control children's sleep. As the father of four, and grandfather of seven, not all that well. But we can control sleep to some extent by making sure that children are tired, and what better way than to have them run around outdoors, etc.

RS: Comparative studies across different regions of the world could be useful in assessing an impact of sunlight, but they should include seasonal and latitude effects, careful attention to lighting variability across the day and parameters related to circadian biology.

FVD: I disagree with the first statement as it is written. The differences may be because those children are not exposed to the light levels that they could have access to. Simply put, children in parts of the world where there is more light may not go outside much. We do not know the answer to that. What we do know is that in certain geographical locations children cannot go outside for reasons related to poverty, e.g., violence. Also, when it is too hot outside people stay indoors. Yes, there is almost certainly more to the effect of outdoors than light levels, but I wanted to point out that we do not even know what light levels children are exposed to just by locating them in a map. Yes, we certainly need to create methodologies that are more robust (and objective) than those currently used to measure the amount of time spent outdoors AND also to measure the actual outdoors visual environment (see question #1).

RC: Yes, absolutely. There are considerable differences in the prevalence of myopia across various regions of the world, and these differences are unlikely to be solely attributed to ethnicity. For e.g., children aged 6-7 with Chinese heritage in Sydney, Australia, have a myopia rate of only 3.3%, while their counterparts in Singapore have a rate of 29% (Rose et al., 2008). The length of the day, or the total daylight hours, also varies significantly depending on the geographic location and time of the year (Lambeck and Cazenave, 1976). For instance, in the month of May, the length of the day ranges from approx.10-12 hours in Australia, 13-16 hours in various parts of the continental USA, about 12 hours in Singapore, and approx. 17-20 hours in different regions of Norway. Again, the length of daylight varies significantly in December, with shorter days in the Northern Hemisphere and longer days in the Southern Hemisphere. The influence of these seasonal variations in daylight on myopia prevalence is not fully understood. Read et al (Read et al., 2018) found that the patterns of daily outdoor light exposure differed significantly between Australia and Singapore. Australian children (105 ± 42 min/d) experienced significantly longer daily outdoor light exposure than Singaporean children (61 ± 40 min/d), with the largest differences found on weekdays during school hours, and these differences may contribute to the differences in myopia prevalence typically found between these populations. There are not many studies directly comparing the daily outdoor light exposure among different populations worldwide. Future studies exploring this research must adopt robust and inclusive methodological approaches and select countries (or populations) based on their latitude and location relative to the equator. These studies should include measurements of outdoor light exposure during various seasons throughout the year, as well as indoor lighting patterns and spectra. Given the recent evidence showing significant changes in axial length and choroidal thickness under various narrowband lighting conditions or spectra (Lou and Ostrin, 2020; Thakur et al., 2021) (including unpublished data from my own lab), the assessment of indoor lighting patterns and spectra is likely a crucial variable. Studies should also consider additional analysis or sub analysis of the data based on weekday vs weekend, ethnicity, parental myopia, near work, educational outcome, sleep patterns etc.

RB. Yes, please see my response above. Currently, we do not have enough data to say that outdoor light levels may not be the predominant factor. A factor that has not been looked at is the quality of the daylight. Although, light levels in Norway are much lower in winter than in countries that are closer to the equator, the quality of the daylight may be higher (and a lower dose may be enough to protect against myopia). Certain types of air pollution are known to reduce the effect of daylight, thus in the most urban cities in the world, the quality of daylight may be very low compared to less urban cities and more rural environments. Also, it may be the combination and variation of outdoor light level and its spectral composition that contributes to healthy regulation of eye growth.

4.- Evidence in animal models show that altered visual input induce changes in retinal/RPE and choroidal expression of **circadian rhythm**-related genes, while an association has been found between a disruption in the sleeping patterns (which alter circadian rhythms) and myopia.

- What studies could be designed to test the desynchronization of the endogenous circadian rhythms likely produced by unnatural lighting patterns and their effect on human myopia?

- Could behavioral or other strategies based on modifying circadian dysregulations be a potential avenue for myopia control?

JW: Short term choroidal thickness studies could be used to examine this. With the evidence on smartphone use before bed affecting circadian rhythm, hence lighting and viewing distance will also be important factors

IM: The problem here is that the dopaminergic and melatonin systems are tightly linked, with increased dopaminergic activity likely to affect circadian rhythms, and disruptions to circadian rhythms likely to affect dopaminergic activity. If there is an association between sleep and myopia, it is because lack of sleep causes myopia, or because children who study late at night have disturbed sleep, but are myopic because they study late at night. Causality is key here. Modifiability is also important. How well can we control children's sleep. As the father of four, and grandfather of seven, not all that well. But we can control sleep to some extent by making sure that children are tired, and what better way than to have them run around outdoors, etc.

RS: The most important studies of circadian dysregulation in myopia development would be conducted in children and young adults, a challenging area for obtaining unambiguous results with minimum bias. Collaboration of eye researchers with sleep and circadian groups would be productive in developing such programs by migrating evolving, state-of-the-art circadian methodology into refractive studies. Important parameters likely would be lighting exposures as a function of time of day, social jet lag and ambient light exposure at night (the latter now being well recognized as an important source of circadian dysregulation, though previously controversial among refraction researchers). Since the anti-myopia optical and drug therapies now being advocated have only modest effects, certainly circadian-related strategies could be studied as potential means for myopia control, either as stand-alone treatments or combined with existing approaches. The field of circadian biology is becoming increasingly complex, and I think that such efforts would be far more likely to be successful if conducted in collaboration with circadian/sleep groups. Further laboratory research, including laboratory research in humans and animals, could be productive; and there are many avenues that could be pursued. While I am not sure of the goal of the National Academy study, I can suggest the following as an unconventional model that might be productive for circadian research. Several years ago, we reported that clock gene disruption in mice induces vitreous chamber elongation and myopia; in Drosophila, clock gene disruption elongates the optical component of ommatidia and seemingly a direct parallel to vertebrate myopia. While our publication never seemed to have gained much traction, studying flies has had such a strong impact on biology and medical research generally, so many well-defined mutants are available, and such powerful imaging techniques are being developed, studying Drosophila as a novel model of refractive research might lead to some innovative insights on circadian effects as well as other potential mechanisms.

FVD: This is another complex area. Dysregulations of circadian rhythms and sleep patterns likely play a role, as indicated by several animal studies. Again, minimal work has been done in humans, and that work has limitations on the techniques to measure circadian rhythms. From these studies we can say that it is likely that behavioral strategies as simple as not using screens after sunset, help with myopia control, but studies are needed.

RC: In recent times, there is a growing interest in understanding the role of circadian dysfunction in myopia pathogenesis (Chakraborty et al., 2018). Whist several studies have reported an association between poor and delayed sleep and childhood myopia, the current evidence is insufficient and conflicting. (Liu et al., 2023). An important concept that is often not discussed is that sleep is not a surrogate for circadian rhythm (CR) or clock measurements. CRs are underlying biological machinery that drive several endogenous rhythms, including the sleep-wake cycle. Therefore, poor sleep doesn't provide information about the underlying biological processes regulating it. The timing of systemic melatonin circadian rhythms is commonly used as a marker for the timing of the endogenous circadian clock. (Claustrat et al., 1995). Unfortunately, even the limited number of studies that have investigated melatonin production, nocturnal onset, and diurnal rhythms in myopic and non-myopic individuals have found inconsistent results. (Hussain et al., 2023). However, currently there is some evidence of circadian rhythm disruption in myopia coming from different labs.(Chakraborty et al., 2020; Flanagan et al., 2020; Kearney et al., 2017; Kumar et al., 2021). Of note, a recent cross-sectional study from Chakraborty's lab at Flinders University found delayed melatonin circadian timing, reduced melatonin production and associated sleep disruptions in myopic children. (Chakraborty et al., 2023). Based on current data, future studies on young children are needed to ascertain whether delays in the melatonin circadian rhythm as well as the preferred timing of sleep and wakefulness during pubertal development are associated with the onset of myopia, and how these factors affect the progression of myopia in childhood. Another approach could be investigating sleep and circadian function in pre-myopic children, to determine if alterations in sleep and circadian rhythms result in early onset and faster progression of myopia in this cohort. These studies should meticulously integrate the investigation of light intensity and spectrum into their methodology, utilizing wearable watches and/or other light devices and sensors, (Hönekopp and Weigelt, 2023) to understand how ambient lighting characteristics may influence circadian rhythms and the subsequent onset and progression of myopia.

Could behavioral or other strategies based on modifying circadian dysregulations be a potential avenue for myopia control? The current evidence linking circadian dysregulation to myopia is rather weak, but is gradually evolving (see review) (Hussain et al., 2023). Whether strategies that could modify circadian rhythms would have any short-term or long-term effect on myopia progression remains to be tested empirically. Given the evidence of delayed sleep and poor sleep quality in myopic children from several cross-sectional and cohort studies, implementing general measures to ensure proper sleep hygiene might benefit overall well-being, including ocular health, and potentially aid in myopia control. Again, carefully designed future studies are warranted to objectively examine sleep characteristics, circadian function, and light exposure patterns in myopic (and pre-myopic) children. This will help understand how these factors predispose the eye to myopia and contribute to its progression. For most organisms, the strongest external environmental stimulus ("zeitgeber") is environmental light (or daily light:dark cycle) that influences circadian rhythm timing and maintains the optimal, stable relationship between biological rhythms and environmental/behavioural interactions (Duffy and Czeisler, 2009). Further research is needed to determine whether altering light exposure patterns in the evening/nighttime (phase-delay region of the phase response curve or PRC) or in the morning/early daytime (phase-advance region of the PRC) (Minors et al., 1991) can be used to modulate circadian rhythm timing and, consequently, myopia in human

RB. First, to my knowledge, there is insufficient evidence to say that there is an association between sleeping patterns and myopia in humans. As with other behavioural measures, there is a need for objective and more accurate measures (and probably measures over some time - a week may not be enough). Field studies utilising sensor that measures activity accurately will also measure in-activity as a proxy for sleep. This may seem a bit superficial to say, but I belive that what is good for general health is good for eye health. Advocating for a healthy lifestyle, would

include advocating for sufficient time outdoors and exposure to daylight (at different times of the day), and this is known to contribute to healthy circadian regulation. More research, however, is required to understand the role of circadian regulation on eye growth. For example, the diurnal rhythm of the crystalline lens appears to natural thicken in the evening (confirmed to be the case also when assessing the effect of atropine on the eye's diurnal rhythm). Activities that require continuous accommodation will contribute to further thickening, but we do not know the consequence of doing near tasks in the evening on the diurnal rhythm of the lens, nor how this may affect the phase relationships between other ocular components of the eye.
